# Time to deterioration of patient-reported outcome endpoints in cancer clinical trials: targeted literature review and best practice recommendations

**DOI:** 10.1186/s41687-024-00824-7

**Published:** 2024-12-18

**Authors:** Kim Cocks, Bellinda L. King-Kallimanis, Joel Sims, Gill Worthy, Julia Stein, Lara Ayala–Nunes, Monika Achra, Zhanglin Lin Cui, Nalin Payakachat

**Affiliations:** 1 Adelphi Values Ltd, Patient-Centered Outcomes, Adelphi Mill, Grimshaw Lane, Bollington, Macclesfield SK10 5JB UK; 2 LUNGevity Foundation, Bethesda, MD USA; 3 Eli Lilly and Company, 639 S. Delaware St, Indianapolis, Indiana 46285 USA

**Keywords:** Patient reported outcome, Neoplasms, Endpoint determination, Survival analysis, Randomized controlled trials, Review literature

## Abstract

**Background:**

Time to deterioration (TTD) endpoints are often utilized in the analysis of patient-reported outcome (PRO) data in oncology clinical trials but different endpoint definitions and analysis frameworks exist that can impact result interpretation. This review examined the analysis, reporting and heterogeneity of TTD endpoints in the literature, the impact of analysis methods on results, and provides recommendations for future trials.

**Methods:**

A targeted literature review of articles published between 2017 and 2022 was performed to collate TTD endpoints reported in oncology randomized controlled trials (RCTs). Details of endpoints and results were extracted including; deterioration definition, PRO assessment schedule, methods for handling intercurrent events, statistical analysis methods, main trial results (overall survival and/or progression-free survival) and TTD endpoint results.

**Results:**

Seventy RCTs were included covering 849 individual TTD endpoints. There were 17 primary cancer types, with lung (26%), breast (11%), and prostate (7%) cancers the most common. Most trials (71%) were for people with advanced cancer. Full definitions of TTD endpoints were often missing. There were no clear trends for a specific TTD definition within cancer types or stages. However, statistical analysis methods were consistent among trials.

**Conclusion:**

The TTD definition can vary and is ultimately driven by the research question. Points to consider for successfully implementing PRO TTD endpoints in oncology include consideration of the trial setting (e.g., early vs. advanced cancer), expected treatment effect (e.g., improvement vs. worsening), likely adverse event profile (including early vs. delayed) and PRO data collection frequency in order to improve utility of these endpoints.

**Supplementary information:**

The online version contains supplementary material available at 10.1186/s41687-024-00824-7.

## Background

Regulators including the Food and Drug Administration (FDA) and European Medicines Agency (EMA) increasingly recognize the importance of using patient-reported outcomes (PROs) in oncology clinical trials for supporting benefit-risk assessment of new therapies [1, 2]. Additionally, health technology assessment (HTA) bodies envision incorporating the patient voice into their reimbursement decisions, which are crucial for patients to have the access to new therapies [3]. Despite overall survival (OS) being universally recognized as the gold standard primary endpoint in anti-cancer trials of new therapies [4–6], PRO endpoints, such as health-related quality of life (HRQoL), are often incorporated. PRO data can be used to investigate clinical benefit and safety, and become especially relevant when the primary endpoint is not OS (e.g., progression-free survival) (PFS)) [7]. PROs are also often used as secondary or exploratory endpoints, and as a co-primary endpoint with OS, PFS, or objective response rate (ORR) [6, 8, 9].

Common analyses of PRO data in oncology clinical trials are time to event (TTE) analyses using Kaplan-Meier methods/Cox proportional hazards regression models or longitudinal analysis using mixed models repeated measures (MMRM) to compare treatment groups [10]. In TTE analyses, the event is either deterioration (time to deterioration (TTD)) or improvement in PRO score from baseline using a pre-specified threshold [11]. TTD is most commonly used as the TTE endpoint. TTD analyses are used to ascertain whether treatment delays worsening in PRO score compared to the control arm. MMRM analyses are used to estimate the mean PRO score (or change from baseline) over a certain period compared between treatment arms [12]. The choice of endpoint and analysis is governed by the clinical question and expectation of the new therapy [13].

The literature reports significant heterogeneity in the definitions used for TTD endpoints. There is a need to harmonize approaches to analyzing and reporting TTD PRO data [7, 14, 15], specifically regarding deterioration definitions and adapting them to different cancer sites and stages (e.g., localized or advanced/metastatic) [14]. A lack of standardization in definitions of TTD, and the resulting lack of comparability across trials have been highlighted by Anota et al. [11] In addition to proposing 36 possible definitions of TTD and time until definitive deterioration (TUDD), the authors suggest developing standardized criteria regarding TTD comparable to criteria used for clinical response definitions [11].

In addition to the lack of standardization in definitions [11] and statistical methods [14], TTD analyses of PRO endpoints share the challenges of the implementation of PRO assessment in clinical trials (e.g., potential high burden to patients due to increased data collection and difficulty maintaining adequate completion rates) as well as its analysis (e.g., missing data handling) [16]. Specific challenges of analyzing TTD endpoints are the difficulty of handling reversibility of the deterioration event (i.e. deterioration at an assessment followed by improvement which may be observed for resolved side effects), the incorporation of intercurrent events (i.e., considering post-randomization events that can affect the interpretation or assessment of patient experience data), and the differing timing of assessments after treatment ends [14, 16].

The goal of this targeted review was to examine how TTD of PRO endpoints have been defined and used in oncology clinical trials (including by specific cancer site and stage), along with how statistical analyses have been reported, using the peer-reviewed literature and regulatory agencies databases.

We then used the literature review data to develop key considerations for using TTD of PRO endpoints in oncology clinical trials, along with associated strengths and weaknesses of different approaches.

## Methods

### Literature search

A targeted literature search of MEDLINE®, Embase®, PsycInfo® and the Cochrane Library was conducted using Ovid in July 2022 (see Appendix A Table A.1 for used search terms), with supplementary searches conducted on ClinicalTrials.gov, EMA/FDA databases and Google Scholar. Reference lists of selected full-text publications, systematic literature reviews (SLR) or network meta-analyses (NMA) were also reviewed.

Inclusion criteria comprised: (i) phase 3 interventional randomized controlled trial (RCT) in oncology with inclusion of a specific PRO measure and TTD endpoint or (ii) guidance, reflection, methods, or review of TTD endpoints in oncology. The period of 2017–2022 was selected based on the seminal publication of Anota et al. [11] in 2015, accounting for a two-year window in the application of the recommended approaches. Conference abstracts and non-English language publications were excluded (Appendix A Table A.1 OVID search terms).

### Screening and selection

Identified abstracts were screened for eligibility (one reviewer per abstract: JSi, LAN, MB); if individual reviewers felt any ambiguity about whether an abstract should be included or excluded, the abstract was discussed among the research team to reach consensus. If two abstracts reported on the same trial, the more detailed reports of PRO analyses were selected, with additional information (e.g., participant baseline data) sourced from the primary trial publications.

Selected abstracts were ranked by amount of TTD information provided to prioritise abstracts with sufficiently detailed presentation of TTD analyses and results to meet the objectives of the study. Ranking was based on information reported in the abstract: (1) TTD hazard ratio (HR) comparing between study treatments and median TTD; (2) TTD HR only; (3) median TTD only; (4) other TTD information. Abstracts that did not report TTD of PRO results (in the form of HR or median TTD) were classed as Rank 4. As Rank 4 articles were expected to be least likely to contain relevant TTD information, they were not selected for full-text review. However, three Rank 4 abstracts were included in the initial extraction of 25 articles used for training and familiarisation with the extraction process (data ultimately being included in the final dataset). Review articles concerning use of TTD of PRO endpoints in oncology were not subject to the ranking process and were all reviewed in full, as they were considered valuable sources of synthesized information of TTD methodologies across trials.

All stages of this targeted review were conducted and reported in line with the Preferred Reporting Items for Systematic Reviews and Meta-Analyses (PRISMA) checklist [17].

### Data extraction

The first ten of the 25 articles were extracted independently by two reviewers and compared to ensure consistency; the remaining 15 articles were extracted by one reviewer. We extracted study information (e.g., cancer type/stage), trial participant clinical and demographic characteristics, PRO assessment schedule (for only PROs contributing to TTD analysis), PRO TTD analysis methods and results, primary efficacy results, and any relationship between efficacy and TTD results. Data were extracted into Microsoft Excel®. Further details of data extraction can be found in Appendix A Table A.2.

### Analysis methods

The characteristics of the included trials were summarized descriptively including number of randomized groups, blinding, primary cancer site, sample size and trial location. Descriptive summaries were presented for the PRO information (measures used in TTD endpoints, schedule of assessments, i.e., cadence of PRO assessments planned) and information on PRO assessments on/off treatment. TTD endpoints were categorised as time to first deterioration (TTFD), time to confirmed deterioration (TTCD) and time until definitive deterioration (TUDD). TTFD refers to a single observed deterioration. TTFD was further grouped into TTFD without death as an event, TTFD with death as an event or TTFD with best previous score (BPS) used as the baseline for deterioration. TTCD refers to a definition requiring two consecutive assessments with a deterioration. TUDD refers to a definition requiring deteriorations observed until no further observations recorded. TUDD was further grouped into TUDD with, or without death as an event. These categorizations alongside the PRO score, the responder definition and reporting of death/progression as event also contributed to how a unique TTD endpoint was defined for each study.

The relationship between TTD and primary endpoints was explored using the proportion of trials demonstrating a treatment effect with respect to TTD PRO endpoints summarized by the trial results for OS and PFS endpoints. Analysis methods and censoring rules for the TTD endpoints were summarized descriptively. No statistical comparisons were performed as part of this review and all results were summarized and interpreted descriptively only.

## Results

**Fig. 1:**
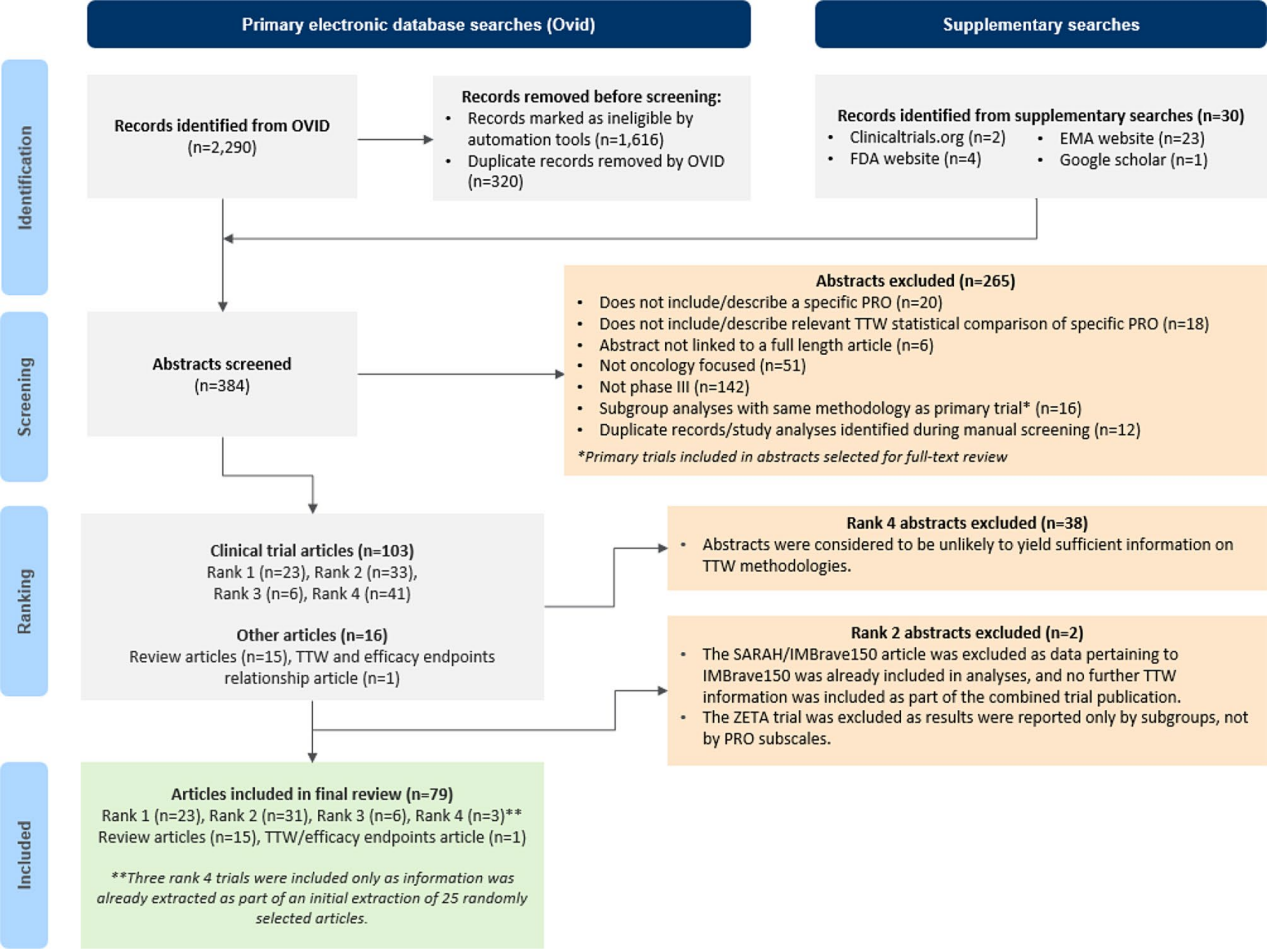
PRISMA [17] diagram illustrating abstract screening and selection process. Rank 1: TTD hazard ratio (HR) and median reported; Rank 2: TTD HR reported only; Rank 3: TTD median reported only; Rank 4: other TTD information reported only

The targeted literature search yielded 384 abstracts (*n* = 354 from electronic database searches; *n* = 30 from supplementary searches) which were screened for eligibility, with 103 clinical trial abstracts and 16 review article abstracts meeting the inclusion criteria. Overall, 79 articles were included for full review (*n* = 63 clinical trial articles, *n* = 1 TTD/efficacy endpoints article, and *n* = 15 review articles). The 63 clinical trial articles reported findings from 70 independent trials, with some pooling data from more than one trial (Fig. 1). Appendix A Table A.3 lists the 70 trials included in the review including their study design, population, and sample size.

### Trial characteristics

Most trials were open-label (*n* = 41/70, 59%) and comprised two arms (*n* = 65/70, 93%). Under half (*n* = 28/70, 40%) were double-blinded (Table 1). There were 17 different primary cancer types. Lung cancer was the most common (*n* = 18/70, 26%), followed by breast (*n* = 8/70, 11%) and prostate cancer (*n* = 5/70, 7%). Most trials were conducted in an advanced/metastatic setting (*n* = 50/70, 71%). The median trial sample size (number of patients randomized) was 564.

**Table 1:** Summary of trial characteristics and PRO assessment schedules included in the review

Characteristic	Number of trials (% of N = 70)
**Cancer site**	
Lung	18 (25.7%)
Breast	8 (11.4%)
Prostate	5 (7.1%)
Kidney	4 (5.7%)
Liver	4 (5.7%)
Multiple Myeloma	4 (5.7%)
Neuroendocrine	4 (5.7%)
Colorectal	3 (4.3%)
Esophageal	3 (4.3%)
Gastric/gastrointestinal stromal	3 (4.3%)
Head & Neck	3 (4.3%)
Lymphoma	3 (4.3%)
Urothelial	3 (4.3%)
Glioblastoma	2 (2.9%)
Biliary Tract	1 (1.4%)
Leukemia	1 (1.4%)
Liposarcoma	1 (1.4%)
**Cancer stage**	
Advanced/metastatic	50 (71.4%)
Non-metastatic	4 (5.7%)
Advanced/metastatic and non-metastatic	7 (10.0%)
Relapsed/refractory	5 (7.1%)
Not reported	4 (5.7%)
**Study design blinding**	
Double-blinded	28 (40.0%)
Open-Label	41 (58.6%)
Partial-blind	1 (1.4%)
**Number of treatment arms**	
Two	65 (92.9%)
Three	4 (5.7%)
Four	1 (1.4%)
**Sample size (total randomized, N)**	
0–199	4 (5.7%)
200–299	6 (8.6%)
300–399	13 (18.6%)
400–499	4 (5.7%)
500–599	10 (14.3%)
600–699	10 (14.3%)
700–799	8 (11.4%)
800–899	4 (5.7%)
900–999	3 (4.3%)
≥1000	8 (11.4%)
**Timing of PRO assessments same across trial arms**	
Yes	66 (94.3%)
No	4 (5.7%)
Not reported	1 (1.4%)
**Initial on-treatment assessment schedule**	
Weekly	4 (5.7%)
Every 2 weeks	3 (4.3%)
Every 3 weeks	18 (25.7%)
Every 4 weeks	17 (24.3%)
Every 6 weeks	10 (14.3%)
Every 8 weeks/Bi-Monthly	10 (14.3%)
Every 12 weeks/3 months	6 (8.6%)
Every 16 weeks	2 (2.9%)
Varied between treatment arms	3 (4.3%)
Not reported	2 (2.9%)
**Initial off-treatment assessment schedule**	
Weekly	2 (4.8%)
Every 2 weeks	2 (4.8%)
Every 3 weeks	16 (38.1%)
Every 4 weeks	10 (23.8%)
Every 6 weeks	4 (9.6%)
Every 8 weeks/Bi-Monthly	4 (9.6%)
Every 12 weeks/3 months	2 (4.8%)
Every 16 weeks	1 (2.4%)
Varied between treatment arms	3 (7.1%)
Not reported	0 (0%)
**On-treatment PRO assessment collection**	
Collected	70 (100%)
Included in TTD Endpoint	70 (100%)
**Off-treatment PRO assessment collection**	
Collected	42 (60%)
Included in TTD Endpoint	15 (21.4%)
Excluded from TTD Endpoint	4 (5.7%)
Not Reported if Included in TTD Endpoint	23 (32.9%)
Not collected	23 (32.9%)
Not reported	5 (7.1%)
**Consistency of on-treatment PRO assessment frequency over the study period**	
Mixed frequency	41 (58.6%)
Same frequency	33 (47.1%)
Not reported	1 (1.4%)

The “Mixed Frequency” option under “Consistency of On-Treatment PRO Assessment Frequency Over the Study Period” consists of trials where the on-treatment frequency changed during the study period (e.g. from every 3 weeks initially to every 4-weeks)

### PRO assessment schedules

Most trials had consistent PRO assessment timing across treatment arms while on-treatment (*n* = 66/70, 94%). There were four trials (CheckMate 227 [18], KEYNOTE-181 [19], KEYNOTE-426 [20] and CheckMate 9ER [21]) where the PRO assessment schedules were not aligned across the treatment arms (due to cycle length differences). All trials used baseline and on-treatment assessments in the PRO TTD analysis. Scheduled off-treatment (i.e. non-protocol treatment) assessments were collected in most of the trials (*n* = 42/70, 60%) but only used in the PRO TTD analysis in 15 trials (*n* = 15/42, 36%). Across trials, the frequency of on-treatment PRO assessments ranged from weekly to every 16 weeks. Half of the trials included on-treatment PRO assessments every three (*n* = 18/70, 26%) or four weeks (*n* = 17/70, 24%).

Most trials had a PRO assessment frequency that was the same as or less regular than the trial dosing schedule (Appendix A Table A.4). Only three trials had a higher PRO assessment frequency compared to dosing [22–24]. Duration of PRO collection was variable with the shortest collection period being 18 weeks [25, 26] and longest up to 260 weeks [27]. Only five trials [25, 26, 28–30] recorded PRO measures for less than 48 weeks. The number of PRO assessments collected ranged from 5 [31] to 57 [21].

TTD endpoints were evaluated using 31 different PRO measures, consisting mostly of cancer-specific PRO measures (Table 2). Most trials included a cancer-specific European Organization for Research and Treatment of Cancer Quality of Life Questionnaire (EORTC QLQ) module. The generic EORTC QLQ-C30 was the most frequently included measure (*n* = 47/70 trials, 67%) and was assessed as a co-primary TTD endpoint in one trial [27] (Table 2). Various EORTC QLQ modules (13 modules) and Functional Assessment of Cancer Therapy (FACT) measures (nine modules) specific to certain cancer types/cancer therapies were commonly utilized for TTD endpoints. Trials were most likely to have at least one exploratory TTD endpoint (*n* = 32/70 trials, 46%) or at least one secondary endpoint (*n* = 30/70, 43%; Table 2.)

**Table 2:** PRO measures used for TTD analysis and endpoint hierarchy

PRO measure	Number of trials (% out of 70 trials)				
	Total*	At least one primary TTD endpoint	At least one secondary TTD endpoint	At least one exploratory TTD endpoint	Not reported
EORTC QLQ modules	54 (77.1%)	1 (1.4%)	22 (31.4%)	23 (32.9%)	11 (15.7%)
EQ-5D	19 (27.1%)	–	5 (7.1%)	11 (15.7%)	3 (4.3%)
FACT	14 (20.0%)	–	7 (10.0%)	7 (10.0%)	1 (1.4%)
BPI-SF	6 (8.6%)	–	2 (2.9%)	3 (4.3%)	2 (2.9%)
LCSS	4 (5.7%)	–	1 (1.4%)	2 (2.9%)	1 (1.4%)
BFI	3 (4.3%)	–	1 (1.4%)	3 (4.3%)	–
MDASI	2 (2.9%)	–	1 (1.4%)	2 (2.9%)	–
NCF battery (HVLT-R, TMT and COWA)	1 (1.4%)	–	-	1 (1.4%)	–
SF-36v2	1 (1.4%)	–	–	1 (1.4%)	–
Skindex-29	1 (1.4%)	–	–	–	1 (1.4%)
Total		1 (1.4%)	30 (42.9%)	32 (45.7%)	13 (18.6%)

*EORTC* European Organisation for Research and Treatment of Cancer, *EQ-5D* EuroQoL Five Dimension, *FACT* Functional Assessment of Cancer Therapy, *BFI* Brief Fatigue Index, *BPI-SF*: Brief Pain Inventory (short form), *SF-36 V2* Short Form 36 Health Survey Questionnaire version 2, *MDASI* MD Anderson Symptom Inventory, *NCF Battery* Neurocognitive Function Battery, *HVLT-R* Hopkins Verbal Learning Test-Revised, *TMT* Trail Making Test, *COWA* Controlled Oral Word Association; *Skindex-29* 29-item Skindex questionnaire

*Some trials reported on more than one TTD PRO endpoint and/or used more than one PRO measure, therefore the total number of endpoints and measures included exceeds the number of trials

Only three trials reported multiple testing adjustments for PRO TTD endpoints; PROFILE 1029 [32] and TITAN [33] made adjustments for TTD of PRO secondary endpoints. NRG/RTOG 0825 [30] made adjustments for tertiary (exploratory) endpoints independent of any other study endpoints. The co-primary endpoint for PRODIGE [27] (with recurrence-free survival) did not report any multiplicity adjustment. In total, *n* = 39/70 trials (56%) reported no multiplicity adjustments across any TTD of PRO endpoint and *n* = 28/70 trials (40%) did not clearly report whether a TTD of PRO endpoint was included in the multiplicity procedure (Table 3).

**Table 3:** Censoring and analysis methods

Study characteristic	Number of trials (% of 70 trials)
**Multiplicity adjustment for PRO TTW endpoint**	
Multiplicity adjustment applied	3 (4.3%)
Multiplicity adjustment not applied	39 (55.7%)
Not reported if multiplicity adjustment applied	28 (40.0%)
**Missing PRO score at any timepoint**	
Missing data handling method reported	27 (38.6%)
Missing data handling method not reported	43 (61.4%)
**Patients with no PRO scores**	
Excluded from the TTD analysis	21 (30.0%)
Censored at randomization	6 (8.6%)
Not reported	43 (61.4%)
**Patients with no baseline PRO scores only**	
Excluded from the TTD analysis	11 (15.7%)
Censored at randomization/Day 1/last assessment	11 (15.7%)
Not reported	48 (68.6%)
**Patients with baseline PRO score only**	
Excluded from the TTD analysis	5 (7.1%)
Censored at randomization/Day 1/last assessment	10 (14.3%)
Not reported	55 (78.6%)
**Missing data/censoring in TTCD and TUDD definitions (applicable in 40 trials)**	
Handling of missing data following initial deterioration (% of 40)	
Censored at last evaluable assessment	1 (2.5%)
Not reported	39 (97.5%)
Initial deterioration occurs at last recorded PRO assessment (% of 40)	
Censored at last evaluable assessment	2 (5.0%)
Considered as deterioration	2 (5.0%)
Not reported	36 (90.0%)
**TTD analysis methods**^**a**^	
Cox proportional hazards model	60 (85.7%)
Log-rank test	45 (64.3%)
Both Cox proportional hazards model and log-rank test	39 (55.7%)
Fine and Gray regression model for competing risks analysis	1 (1.4%)
Not reported	3 (4.3%)

^**a**^**TTD Analysis Methods:** Categories are not mutually exclusive

### PRO TTD endpoint definitions

There were 849 TTD endpoints identified in total when counting the number of endpoint definitions across all trials. The average number of TTD endpoints per trial was 12 (median = 7). Four multi-arm trials applied the same TTD endpoint definition to more than one treatment arm comparison generating 913 unique endpoint definition results across all trials and treatment comparisons.

The included trials reported all three definitions of TTD (TTFD, TTCD and TUDD) across the primary cancer types identified Appendix Table A.5) TTFD was reported across most cancer types (14/17 cancers). All three TTD definitions were utilised across lung, breast, prostate, kidney, and liver cancer trials. Trials assessing multiple myeloma (*n* = 4), lymphoma (*n* = 3) and leukaemia (*n* = 1) only reported TTFD endpoints.

TTFD analyses were reported by trials of different cancer stages (advanced/metastatic, non-metastatic, both advanced/metastatic and non-metastatic and relapsed/refractory). No trials of relapsed/refractory cancer reported TTCD or TUDD analyses (Appendix Table A.5). Some trials included more than one TTD definition. Altogether, TTFD was the most common definition identified in over half of the included trials (*n* = 38/70 trials, 54%), followed by TTCD (*n* = 21/70, 30%) and TUDD (*n* = 19/70, 27%).

Five distinct TTD definitions described by Anota et al. [11] were identified in the review, including TTFD without death as an event, TTFD with death as an event, TTFD with death as an event using BPS, TUDD without death as an event and TUDD with death as an event. As previously noted, TTCD, was also identified as an additional definition (Appendix Table A.6). The most common event definition was TTFD without death as an event (using baseline as reference score), identified in *n* = 30/70 (43%) trials. Other common TTD event definitions included TTCD and TUDD without death as an event (using baseline as reference score). Notably, fewer trials were identified which included death and/or disease progression as an event, with more trials using death (*n* = 21/70, 30%) than disease progression (*n* = 11/70, 16%). Four trials (6%) included both death and progression in the event definition. One trial used a reference score other than baseline (NETTER-1) [34], using TTFD with death as an event calculated from BPS, as a sensitivity analysis (Appendix Table A.6).

Post-progression assessments were collected in *n* = 27/70 (39%) of the trials. When post-progression assessments were collected, they were included in the TTD endpoint definition for less than half of those trials (*n* = 12/27 trials, 44%), although a similar number had unclear reporting of their inclusion (*n* = 13/27, 48%). Two trials collected post-progression assessments but did not include these assessments in the TTD endpoint analysis nor did they consider disease progression as a TTD event.

### TTD of PRO endpoint censoring rules

Overall, *n* = 43/70 (61%) trials did not report how patients with missing PRO scores at any time point were incorporated into the analysis (five of which reported no imputation conducted) [35]. Patients with no PRO scores were either excluded from analysis in *n* = 21/70 (30%) of the trials or were censored at randomization in six trials [18, 20, 31, 34, 36, 37] (9%). A large proportion of trials did not report how patients with no baseline PRO scores but with subsequent post baseline assessments were handled in the analysis (*n* = 48/70, 69%). Eleven trials [26, 31, 32, 38–45] excluded such patients from the TTD analysis and the same number censored these patients at randomization/day 1/last assessment (*n* = 11/70, 16%). Similarly, most trials did not report how patients who completed only a baseline PRO were incorporated into the analysis (*n* = 55/70, 79%). When this information was reported, patients were either excluded from the analysis in five trials [26, 32, 38, 40, 43] or were included and censored at randomization/Day 1 in another *n* = 10/70 (14%) trials (Table 3).

For the 40 trials using either TTCD or TUDD definitions of TTD, only one trial (ADAURA) [46] reported a missing data rule for visits following the initial deterioration (patients with two missed visits before confirmed deterioration were censored at the last evaluable assessment before the two missed visits). Only four of the trials using TTCD/TUDD explicitly stated whether a deterioration at the last recorded PRO assessment was considered as a confirmed/definitive deterioration: PROSPER [47] and JAVELIN [48] censored such patients; CheckMate 9ER [21] and CLEAR [41] considered these patients to have confirmed and definitive deterioration, respectively (Table 3).

### Composite PRO TTD endpoints

Composite endpoints formulated as composites of multiple PRO assessments or with other clinical endpoints were identified in *n* = 10/70 trials (14%; excluding composites of death and disease progression). The most common was the composite of dyspnea, cough, and chest pain items of the FACT-L as reported in six trials (an event was deterioration in any one item). Most composites consisted only of PRO measures, however two trials (MONARCH [49] and TITAN [33]) used increase in opiate usage (or pain progression) to define a deterioration event. Patient drop-out was included in the definition of deterioration (alongside death and disease progression) in one trial OTT 0101/RTOG 9413 [44].

### PRO TTD analysis methods

The most common statistical methods for the TTD analyses included the Cox proportional hazards model (*n* = 60/70, 86%), and the log-rank test (*n* = 45/70, 64%). More than half the trials reported using both (*n* = 39/70, 56%). Separately, *n* = 21/70 (30%) of trials only reported using the Cox proportional hazards model and *n* = 6/70 (9%) of trials only reported using the log-rank test. It was not clearly reported in three trials (*n* = 3/70, 4%) whether a log-rank and/or Cox proportional hazards model was used. The only trial not to use either a log-rank test or Cox model was Morabito et al. [25] describing the competing risk analyses of data from MILES-3/MILES-4 using a Fine and Gray regression model (Table 3).

Regarding TTD endpoint analyses, 13% (*n* = 9/70) of the trials did not state which factors were included in the statistical model. One trial (REFLECT [45]) stated that there were no factors included in the TTD statistical model and eight trials [29, 32, 34, 42, 46, 50–52] explicitly stated that the TTD statistical model was unstratified (ADAURA [46] justified this as a result of low event counts). Half (*n* = 35/70) of the trials explicitly stated that the TTD endpoint analyses were stratified and *n* = 19/70 (27%) listed the stratification factors used. Three (CheckMate 141 [53], CheckMate 274 [54] and MILES-3/MILES-4 [25]) trials stated that the baseline PRO value was used as a covariate. Most trials (*n* = 63/70, 90%) did not report the method used for tied survival times. Of those that did, six used the Efron method and one trial (MYSTIC [22]) used the Breslow approach [55].

Most trials (*n* = 43/70, 61%) did not report any sensitivity analysis for TTD. Overall, *n* = 27/70 (39%) reported incorporating some sensitivity analysis for the PRO TTD endpoint. The most common involved testing a different responder definition (RD) threshold used to define a deterioration (*n* = 8/27, 30%).

### PRO TTD endpoint analysis results

**Fig. 2:**
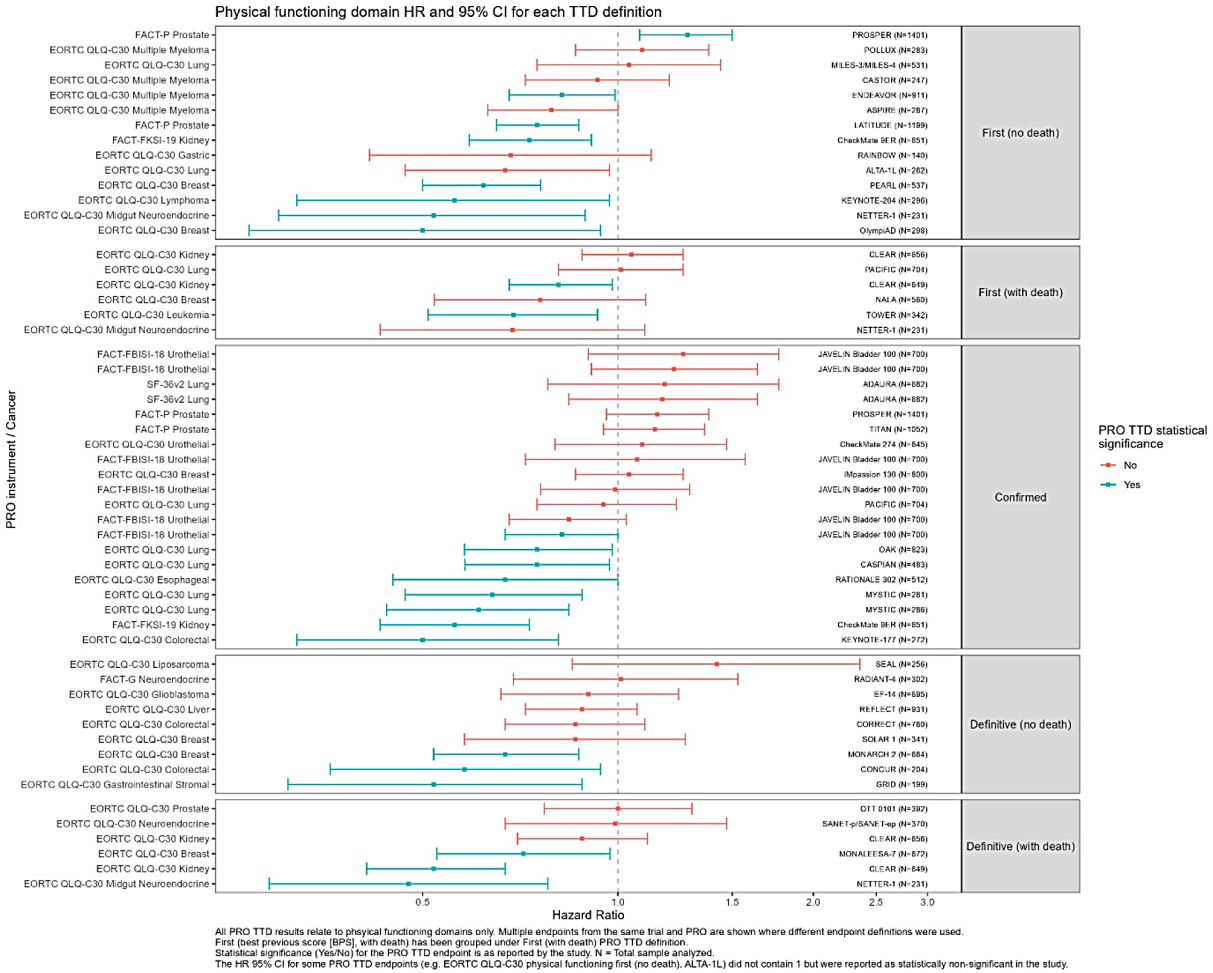
Physical functioning domains hazard ratios reported for each TTD definition

Figure 2 shows the PRO TTD results comparing treatment arms (HR and 95% confidence intervals) for physical functioning specific TTD endpoints. Physical functioning endpoints were selected since this was the most commonly reported domain. The results are displayed by TTD definition. The range of HR is broadly similar regardless of the definition and statistically significant results were observed for each definition.

Most TTD of PRO endpoints yielded between 20 and 60% of patients with events (Appendix Figure A.1). The higher proportion of patients experiencing TTD events and higher proportion of statistically significant PRO TTD endpoints were seen for TTFD, as might be expected since worsening at only one timepoint is required and therefore both transient and non-transient events are captured by the endpoint. It should be noted though that there are trials where TTCD and TUDD definitions also have relatively high proportions (40–80%) of patients with events and statistically significant TTD of PRO endpoints. Despite some trials having low power with only 10–20% of patients with TTD events, there were still some statistically significant results observed across the TTD definitions (Appendix Figure A.1).

Across all TTD definitions, there were both statistically significant and non-significant results. Appendix Figure A.2 shows that within cancer sites, various TTD definitions were used. Some cancer sites (e.g., gastric/GI, prostate, neuroendocrine) consistently had no (or relatively very few) differences between treatment arms for TTD endpoints. Within some cancer sites, some TTD definitions had higher proportions of statistically significant results compared with others, for example in breast cancer, TTFD (no death) and TUDD (with death) had a numerically higher proportion of significant results compared to TTFD (with death) and TUDD (no death). There was no consistent pattern in other cancer sites. Within cancer sites, a higher frequency of events was not always associated with a higher proportion of statistically significant PRO TTD endpoints.

### Relationship between efficacy endpoints and PRO TTD endpoint results

**Fig. 3:**
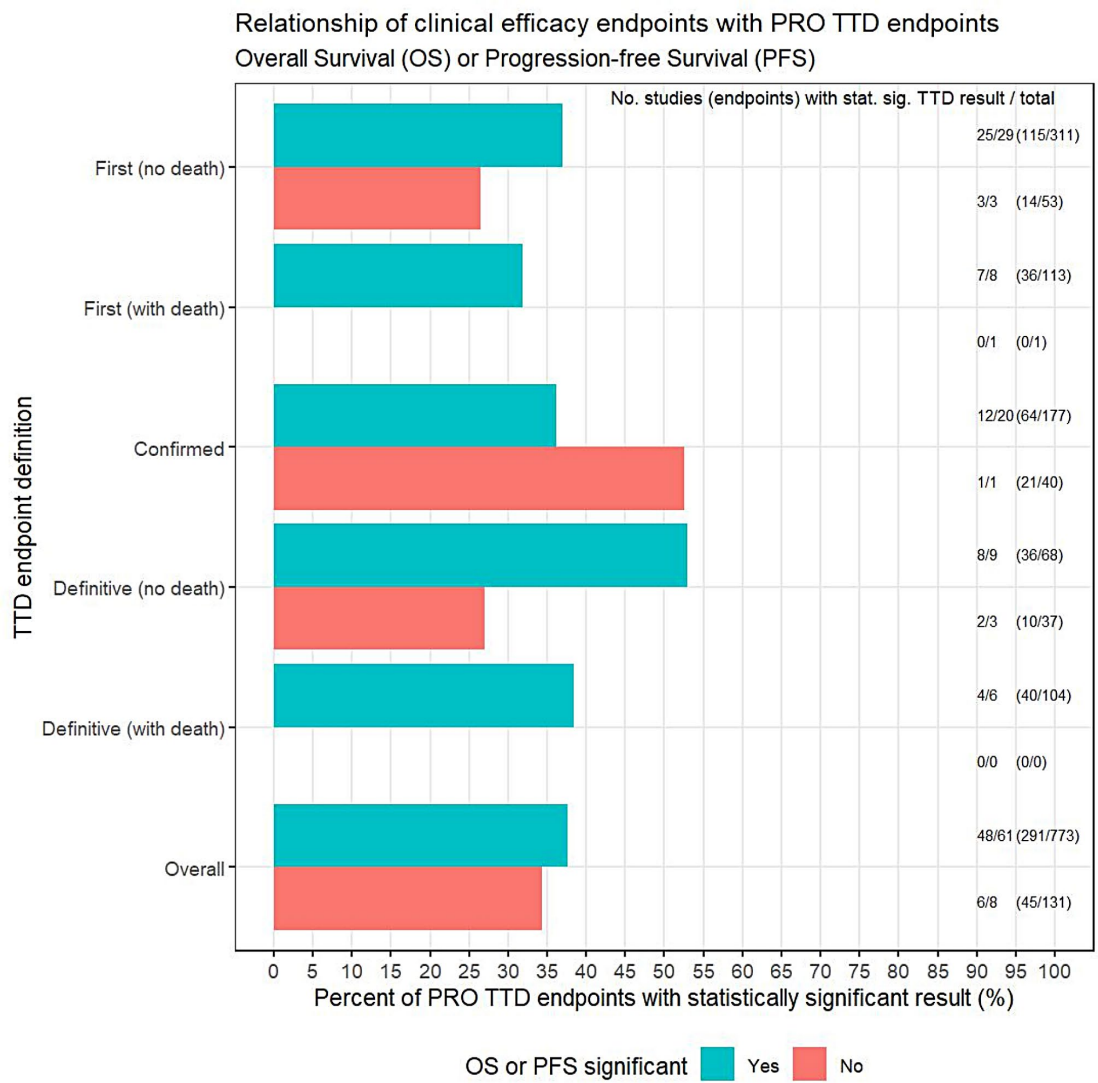
Proportion of patients with events by trial and OS/PFS significance. Number of endpoints represents the denominator for percentage calculation (specifically number of endpoints within each TTD endpoint definition and OS/PFS result). Studies without an associated OS or PFS result were excluded from the analysis (*n* = 1 trial consisting of 9 TTD endpoints)

Figure 3 illustrates a potential relationship between the efficacy results (statistically significant OS or PFS) and the reporting of at least one statistically significant TTD endpoint. Overall, 38% (291/773) of PRO TTD endpoints from studies showing treatment benefit in OS or PFS were statistically significant endpoints. The TTD definition did not appear to affect this relationship although the number of studies reporting no significant OS/PFS result was small (*n* = 8/70, 11%).

## Discussion

This review identified that the current PRO TTD endpoints in oncology clinical trials are heterogeneous with respect to the definitions used. Differences in definitions and analysis frameworks were not explained by differences in trial characteristics, which would indicate that within cancer sites and stages there is no consensus on the most appropriate definition. The lack of clear reporting of the full analysis framework however limited the extent to which this could be explored. Descriptively, there was no clear association between definitions or analysis methods and whether there was a statistically significant result for the PRO TTD endpoint, indicating that there should not be a preference based on the perceived likelihood of ‘success’ of a particular definition. The driver for choice of endpoint should be careful consideration of the research question and selection of an appropriate estimand framework to answer that question. For example, TTCD could be perceived as a riskier endpoint strategy since there may be more missing data as it requires having data on at least two consecutive assessments. It also means observing fewer events than TTFD since it removes transient deteriorations from the endpoint, therefore potentially reducing the statistical power. Our findings however indicate that TTCD and TUDD endpoints can yield sufficient events in the appropriate setting.

Our review included 70 oncology trials. Although there was heterogeneity in TTD definitions, there was a high degree of consistency in the use of statistical analysis methods. However, many trials did not fully define the TTD endpoint, and this could be improved by following existing guidance for including PRO endpoints in protocols (SPIRIT-PRO) [56], specification of estimands [57] and reporting of PRO data (CONSORT-PRO) [58].

Using the ICH E9 R1 estimand framework [59], all elements of a TTD endpoint would be presented. Firstly, a clear objective for the PRO endpoint should be defined. This is often overlooked yet it forms the basis for an adequately defined endpoint [60]. Objectives will vary across oncology trials depending on the disease (e.g., an advanced prostate cancer trial compared to an advanced pancreatic cancer trial) and treatment (1st line vs 2nd line therapy), therefore, variation in the attributes of the estimand is inevitable. There may be some settings where time to improvement PRO endpoints are more appropriate, for example in an adjuvant setting measuring PROs post-surgery where improvement in the long term is anticipated.

Within the estimand framework, a clearly specified objective informs the most appropriate intercurrent event handling strategy, which in turn helps with the planning of the PRO assessment schedule. More frequent PRO assessments, or inclusion of post-progression assessments may be required to facilitate a TTD analysis where treatment duration or prognosis is short. For some studies, TTD of a PRO endpoint may not be feasible if there are a limited number of PRO assessments planned. A treatment policy strategy (where the interest lies in the treatment effect regardless of intercurrent event occurrence) may be desirable in many situations but would require assessments continuing after disease progression or after discontinuing treatment. This requires planning to ensure feasibility and quality of data collection after treatment discontinuation. Even if a while-on-treatment strategy was used, ultimately, if the PRO schedule is not logistically practical then it is likely there will be high amounts of missing data. Therefore, the choice of estimand may be limited by implementation of the PRO assessment schedule in the trial and the two need to be considered in parallel.

There are examples of TTD endpoints being included in prescription drug labelling. For instance, TTD endpoint results for the patient-reported concepts of “shortness of breath” (FDA) and cough, pain and dyspnea (EMA) were included in the prescribing/product information for ceritinib (Zykadia®), approved for patients with metastatic anaplastic lymphoma kinase (ALK)-positive Non-small cell lung cancer (NSCLC) [61, 62]. However the point estimate for this TTD endpoint was not included in the FDA label due to its positioning as an exploratory endpoint. The elevation of a TTD endpoint to a key secondary endpoint and placement within the testing hierarchy, for example, may be especially pertinent in advanced settings where delaying worsening of symptoms may be highly valued by patients. With novel treatments, little may be known about the expected impact on PRO data, and clear hypotheses may not be feasible leading to these endpoints being specified as exploratory. Consideration of the expected course of the disease and treatment intentions should lead to an improvement in both the definition of use of TTD endpoints.

A strength of this review is that the extracted data from the current literature can be used to facilitate planning for future trial endpoints. With respect to planning the PRO assessment schedule, we found that most studies link the PRO assessment schedule to the study dosing schedule (day 1 of a treatment cycle). However, we identified examples where the dosing schedules differed between treatment groups (e.g. 3-weekly vs 4-weekly cycles), which can lead to analytical challenges. Other challenges are incorporating an appropriate PRO assessment schedule for oral (potentially daily dosing) versus intravenous treatments (e.g., administered on alternate days in the first week followed by a treatment break during a 3-week cycle). PRO schedules on the first day of dosing may capture the worst side effects on day 1 for the intravenous (IV) group but miss cumulative side effects from continued daily dosing. Historically PRO assessments in cancer clinical trials were linked to clinic visits to increase completion rates, however, by decoupling PRO assessments with cycle visits, it is possible to increase our understanding of how symptoms change over the course of treatment.

With respect to PRO TTD endpoint definition, we found that most trials had inadequate reporting (e.g., not defining the deterioration event in sufficient detail or how intercurrent events were handled). Although there was a lack of consistency with respect to the PRO TTD endpoint definition across included trials, this may in part be due to the differing underlying objectives but also due to the categorizing trials by cancer type and stage. It is possible that within the broad categorizations there are different patient populations and different treatment goals under investigation and therefore different PRO TTD endpoint definitions were justified. This highlights that the expectation for a single consistent TTD of PRO definition may not be realistic but, aligned with Anota et al. [11] a consensus could potentially be achieved within specific settings. The choice of definition is also impacted by missing data. For example, more missing data may be observed with TTCD compared with TTFD, since there is a requirement for deterioration in consecutive PRO assessments. In an advanced disease setting it may be unrealistic to plan a TTCD/TUDD endpoint even if it were desirable to consider definitive deterioration rather than possibly transient changes.

With respect to results for PRO TTD endpoints, this review provides a unique opportunity to see the impact of the different definitions on number of deterioration events observed and the impact on statistical power for the study. These data can support researchers in planning and evaluating PRO TTD endpoints for future studies. It also highlights the value of these endpoints in supporting primary endpoint results, such as OS and PFS.

Limitations of this targeted review include the extent of the search strategy, and review methods compared those used in systematic literature reviews. Following execution of the searches, we ranked the abstracts to select the most relevant articles for data extraction, favouring those with better reporting of TTD of PRO results in the abstract. While this may have limited the number of trials from which TTD data was extracted, it is unlikely that the conclusions of this review would differ. It is possible that the inclusion of abstracts with limited TTD reporting may have further strengthened our recommendation for better reporting of TTD definitions. The results of this review provide an overview of findings from TTD endpoints, which has been previously reported, though in a smaller pool of 39 Phase III studies [14]. The Charlton et al. review concluded that attention should be paid to the definition of deterioration and that this should be based on the specific cancer setting. The motivation for the current review and for summarising data from TTD endpoints by cancer and stage therefore was to facilitate consideration of how the definition may vary by cancer setting. This targeted review also provides further analysis and critique of TTD of PRO endpoints from a wider range of trials and consolidates these results with the existing literature.

Our key recommendations include carefully defining the TTD endpoint using the estimand framework and considering the trial setting and expected treatment effect. The nuances of how TTD is ultimately defined links to a different research objective and estimand. This will be true within cancer types and stages. This may explain the lack of consistency observed across trials. Rather than focusing on achieving consistency in definitions of TTD endpoints, our recommendations are to focus on improved clarity of the TTD objective and endpoint, and considering the assumptions required to conduct a survival analysis of these data.

## Conclusion

TTD endpoints are one type of endpoint for the demonstration of treatment benefit using PRO data alongside other endpoints associated with changes in mean score over time and responder-based analyses. The choice of endpoint is ultimately led by the research question. Although the analysis of PRO TTD endpoints has challenges to address, the results can be used to help guide regulators when clinical benefit magnitude is small, and they could help facilitate decision making between patients and oncologists when more than one treatment option is available. Following existing guidelines for PRO endpoint planning in protocols (SPIRIT-PRO) [56], estimand definition [57, 59] and reporting of PRO analyses (CONSORT PRO extension) [58] will improve their utility and impact.

## Electronic supplementary material

Below is the link to the electronic supplementary material.

Supplementary Material 1

## Data Availability

All data generated or analysed during this study are included in this published article and its supplementary information files.

## Abbreviations

ALK: Anaplastic Lymphoma Kinase
BFI: Brief Fatigue Index
BPI-SF: Brief Pain Inventory (short form)
BPS: Best previous score
EMA: European Medicines Agency
EORTC QLQ: European Organization for Research and Treatment of Cancer Quality of Life Questionnaire
EQ-5D: EuroQoL Five Dimension
FACT: Functional Assessment of Cancer Therapy
FDA: Food and Drug Administration
HR: Hazard ratio
HRQoL: Health-related quality of life
HTA: Health technology assessment
HVLT-R: Hopkins Verbal Learning Test-Revised
IV: Intravenous
LCSS: Lung Cancer Symptom Scale
MDASI: MD Anderson Symptom Inventory
MMRM: Mixed models repeated measures
NCF Battery: Neurocognitive Function Battery
NMA: Network meta-analyses
NSCLC: Non-small cell lung cancer
ORR: Objective response rate
OS: Overall survival
PFS: Progression-free survival
PRISMA: Preferred Reporting Items for Systematic Reviews and Meta-Analyses
PRO: Patient reported outcome
RCT: Randomized controlled trials
RD: Responder definition
SF-36 V2: Short Form 36 Health Survey Questionnaire version 2
Skindex-29: 29-item Skindex questionnaire
SLR: Systematic literature reviews
TTCD: Time to confirmed deterioration
TTD: Time to deterioration
TTE: Time to event
TTFD: Time to first deterioration
TUDD: Time until definitive deterioration

## References

[1] Meregaglia M, Malandrini F, Angelini S, Ciani O (2023) The assessment of patient-reported outcomes for the authorisation of medicines in Europe: a review of European Public Assessment Reports from 2017 to 2022. Appl Health Econ Health Policy 21(6):925–935 37659000 10.1007/s40258-023-00827-3 PMC10627987

[2] Food and Drug Administration (FDA) Enhancing the incorporation of the patient’s voice in medical product development and regulatory decision making. https://www.fda.gov/drugs/development-approval-process-drugs/fda-patient-focused-drug-development-guidance-series-enhancing-incorporation-patients-voice-medical

[3] Chassany O, Engen A, Lai L et al (2022) A call to action to harmonize patient-reported outcomes evidence requirements across key European HTA bodies in oncology. Future Oncol 18(29):3323–3334 36053168 10.2217/fon-2022-0374

[4] Han K, Ren M, Wick W et al (2014) Progression-free survival as a surrogate endpoint for overall survival in glioblastoma: a literature-based meta-analysis from 91 trials. Neuro-oncology 16(5):696–706 24335699 10.1093/neuonc/not236 PMC3984546

[5] Beauchemin C, Johnston J, Lapierre M, Aissa F, Lachaine J (2015) Relationship between progression-free survival and overall survival in chronic lymphocytic leukemia: a literature-based analysis. Current Oncol 22(3):148–156 10.3747/co.22.2119 PMC4462536 26089725

[6] Food and Drug Administration (FDA) (2018) Clinical trial endpoints for the approval of cancer drugs and biologics - guidance for industry

[7] Marandino L, De Luca E, Zichi C et al (2019) Quality-of-life assessment and reporting in prostate cancer: systematic review of phase 3 trials testing anticancer drugs published between 2012 and 2018. Clin Genitourin Cancer 17(5):332–347.e2 31416754 10.1016/j.clgc.2019.07.007

[8] Kilickap S, Demirci U, Karadurmus N, Dogan M, Akinci B, Sendur MAN (2018) Endpoints in oncology clinical trials. J Buon 23(7):1–6 30722104

[9] Fiteni F, Pam A, Anota A et al (2015) Health-related quality-of-life as co-primary endpoint in randomized clinical trials in oncology. Expert Rev Anticancer Ther 15(8):885–891 26027598 10.1586/14737140.2015.1047768

[10] Fiero MH, Roydhouse JK, Vallejo J, King-Kallimanis BL, Kluetz PG, Sridhara R (2019) US Food and Drug Administration review of statistical analysis of patient-reported outcomes in lung cancer clinical trials approved between January, 2008, and December, 2017. Lancet Oncol 20(10):e582–e589. 10.1016/S1470-2045(19)30335-3 31579004 10.1016/S1470-2045(19)30335-3

[11] Anota A, Hamidou Z, Paget-Bailly S et al (2015) Time to health-related quality of life score deterioration as a modality of longitudinal analysis for health-related quality of life studies in oncology: do we need RECIST for quality of life to achieve standardization? Qual Life Res 24(1):5–18 24277234 10.1007/s11136-013-0583-6 PMC4282717

[12] Machingura A, Coens C, Pe M et al. Methodological work and use of patient-reported outcomes data in Randomised Controlled Trials (Rcts) in cancer: literature reviews on current practices and guidelines

[13] Mercieca-Bebber R, King MT, Calvert MJ, Stockler MR, Friedlander M (2018) The importance of patient-reported outcomes in clinical trials and strategies for future optimization. Patient Relat Outcome Meas 9:353–367 30464666 10.2147/PROM.S156279 PMC6219423

[14] Charton E, Cuer B, Cottone F et al (2020) Time to deterioration in cancer randomized clinical trials for patient-reported outcomes data: a systematic review. Qual Life Res 29(4):867–878 31776827 10.1007/s11136-019-02367-7

[15] Bascoul-Mollevi C, Barbieri A, Bourgier C et al (2021) Longitudinal analysis of health-related quality of life in cancer clinical trials: methods and interpretation of results. Qual Life Res 30(1):91–103 32809099 10.1007/s11136-020-02605-3

[16] Fiero MH, Roydhouse JK, Bhatnagar V et al (2022) Time to deterioration of symptoms or function using patient-reported outcomes in cancer trials. Lancet Oncol 23(5):e229–e234 35489354 10.1016/S1470-2045(22)00021-3

[17] Page MJ, McKenzie JE, Bossuyt PM et al (2021) The PRISMA 2020 statement: an updated guideline for reporting systematic reviews. BMJ 372:n71. 10.1136/bmj.n71 33782057 10.1136/bmj.n71 PMC8005924

[18] Reck M, Ciuleanu T-E, Lee J-S et al (2021) First-line nivolumab plus ipilimumab versus chemotherapy in advanced NSCLC with 1% or greater tumor PD-L1 expression: patient-reported outcomes from CheckMate 227 Part 1. J Thorac Oncol 16(4):665–676 33485960 10.1016/j.jtho.2020.12.019

[19] Adenis A, Kulkarni AS, Girotto GC et al (2022) Impact of pembrolizumab versus chemotherapy as second-line therapy for advanced esophageal cancer on health-related quality of life in KEYNOTE-181. J Clin Oncol 40(4):382–391 34730989 10.1200/JCO.21.00601

[20] Bedke J, Rini BI, Plimack ER et al (2022) Health-related quality of life analysis from KEYNOTE-426: pembrolizumab plus axitinib versus sunitinib for advanced renal cell carcinoma. Eur Urol 82(4):427–439 35843776 10.1016/j.eururo.2022.06.009

[21] Cella D, Motzer RJ, Suarez C et al (2022) Patient-reported outcomes with first-line nivolumab plus cabozantinib versus sunitinib in patients with advanced renal cell carcinoma treated in CheckMate 9ER: an open-label, randomised, phase 3 trial. Lancet Oncol 23(2):292–303 35032437 10.1016/S1470-2045(21)00693-8 PMC9479564

[22] Garon EB, Cho BC, Reinmuth N et al (2021) Patient-reported outcomes with durvalumab with or without tremelimumab versus standard chemotherapy as first-line treatment of metastatic non–small-cell lung cancer (MYSTIC). Clin Lung Cancer 22(4):301–312.e8 33775558 10.1016/j.cllc.2021.02.010

[23] Lee CK, Novello S, Rydén A et al (2018) Patient-reported symptoms and impact of treatment with osimertinib versus chemotherapy in advanced non-small-cell lung cancer: the AURA3 trial. J Clin Oncol 36(18):1853–1860 29733770 10.1200/JCO.2017.77.2293

[24] Atkins MB, Rini BI, Motzer RJ et al (2020) Patient-reported outcomes from the Phase III randomized IMmotion151 trial: atezolizumab+ Bevacizumab versus sunitinib in treatment-naïve metastatic renal cell CarcinomaIMmotion151 PROs with Atezolizumab plus Bevacizumab in mRCC. Clin Cancer Res 26(11):2506–2514 32127394 10.1158/1078-0432.CCR-19-2838 PMC8407399

[25] Morabito A, Piccirillo MC, Maione P et al (2019) Effect on quality of life of cisplatin added to single-agent chemotherapy as first-line treatment for elderly patients with advanced non-small cell lung cancer: joint analysis of MILES-3 and MILES-4 randomised phase 3 trials. Lung Cancer 133:62–68 31200830 10.1016/j.lungcan.2019.05.009

[26] Van Cutsem E, Kato K, Ajani JA et al (2022) Tislelizumab versus chemotherapy as second-line treatment for advanced or metastatic esophageal squamous cell carcinoma (ESCC, RATIONALE 302): impact on health-related quality of life (HRQoL). Am Soc Clin Oncol 10.1016/j.esmoop.2022.100517 PMC9434166 35785595

[27] Edeline J, Benabdelghani M, Bertaut A et al (2019) Gemcitabine and oxaliplatin chemotherapy or surveillance in resected biliary tract cancer (PRODIGE 12-ACCORD 18-UNICANCER GI): a randomized phase III study. J Clin Oncol 37(8):658–667 30707660 10.1200/JCO.18.00050

[28] Bordoni R, Ciardiello F, von Pawel J et al (2018) Patient-reported outcomes in OAK: a phase III study of atezolizumab versus docetaxel in advanced non–small-cell lung cancer. Clin Lung Cancer 19(5):441–449.e4 30017645 10.1016/j.cllc.2018.05.011

[29] Andre T, Amonkar M, Norquist JM et al (2021) Health-related quality of life in patients with microsatellite instability-high or mismatch repair deficient metastatic colorectal cancer treated with first-line pembrolizumab versus chemotherapy (KEYNOTE-177): an open-label, randomised, phase 3 trial. Lancet Oncol 22(5):665–677 33812497 10.1016/S1470-2045(21)00064-4

[30] Wefel JS, Armstrong TS, Pugh SL et al (2021) Neurocognitive, symptom, and health-related quality of life outcomes of a randomized trial of bevacizumab for newly diagnosed glioblastoma (NRG/RTOG 0825). Neuro-oncology 23(7):1125–1138 33515019 10.1093/neuonc/noab011 PMC8661434

[31] Taphoorn MJ, Dirven L, Kanner AA et al (2018) Influence of treatment with tumor-treating fields on health-related quality of life of patients with newly diagnosed glioblastoma: a secondary analysis of a randomized clinical trial. JAMA Oncol 4(4):495–504 29392280 10.1001/jamaoncol.2017.5082 PMC5885193

[32] Wu Y-L, Lu S, Lu Y et al (2018) Results of PROFILE 1029, a phase III comparison of first-line crizotinib versus chemotherapy in East Asian patients with ALK-positive advanced non–small cell lung cancer. J Thorac Oncol 13(10):1539–1548 29966800 10.1016/j.jtho.2018.06.012

[33] Agarwal N, McQuarrie K, Bjartell A et al (2019) Health-related quality of life after apalutamide treatment in patients with metastatic castration-sensitive prostate cancer (TITAN): a randomised, placebo-controlled, phase 3 study. Lancet Oncol 20(11):1518–1530 31578173 10.1016/S1470-2045(19)30620-5

[34] Strosberg J, Wolin E, Chasen B et al (2018) Health-related quality of life in patients with progressive midgut neuroendocrine tumors treated with 177Lu-dotatate in the phase III NETTER-1 trial. J Clin Oncol 36(25):2578 29878866 10.1200/JCO.2018.78.5865 PMC6366953

[35] Hofheinz R-D, Bruix J, Demetri GD et al (2021) Effect of regorafenib in delaying definitive deterioration in health-related quality of life in patients with advanced cancer of three different tumor types. Cancer Manage Res 13:5523 10.2147/CMAR.S305939 PMC8285228 34285574

[36] Pérol M, Pavlakis N, Levchenko E et al (2019) Patient-reported outcomes from the randomized phase III ALEX study of alectinib versus crizotinib in patients with ALK-positive non-small-cell lung cancer. Lung Cancer 138:79–87 31654838 10.1016/j.lungcan.2019.10.002

[37] Porcu P, Hudgens S, Horwitz S et al (2021) Quality of life effect of the anti-CCR4 monoclonal antibody mogamulizumab versus vorinostat in patients with cutaneous T-cell lymphoma. Clin Lymphoma Myeloma Leukemia 21(2):97–105 10.1016/j.clml.2020.09.003 PMC7878351 33158772

[38] Campelo MRG, Lin HM, Zhu Y et al (2021) Health-related quality of life in the randomized phase III trial of brigatinib vs crizotinib in advanced ALK inhibitor–naive ALK+ non− small cell lung cancer (ALTA-1L). Lung Cancer 155:68–77 33744781 10.1016/j.lungcan.2021.03.005

[39] Goldman JW, Garassino MC, Chen Y et al (2020) Patient-reported outcomes with first-line durvalumab plus platinum-etoposide versus platinum-etoposide in extensive-stage small-cell lung cancer (CASPIAN): a randomized, controlled, open-label, phase III study. Lung Cancer 149:46–52 32961445 10.1016/j.lungcan.2020.09.003

[40] Scherpereel A, Antonia S, Bautista Y et al (2022) First-line nivolumab plus ipilimumab versus chemotherapy for the treatment of unresectable malignant pleural mesothelioma: patient-reported outcomes in CheckMate 743. Lung Cancer 167:8–16 35367910 10.1016/j.lungcan.2022.03.012

[41] Motzer R, Porta C, Alekseev B et al (2022) Health-related quality-of-life outcomes in patients with advanced renal cell carcinoma treated with lenvatinib plus pembrolizumab or everolimus versus sunitinib (CLEAR): a randomised, phase 3 study. Lancet Oncol 23(6):768–780 35489363 10.1016/S1470-2045(22)00212-1 PMC10284118

[42] Leighl NB, Karaseva N, Nakagawa K et al (2020) Patient-reported outcomes from FLAURA: osimertinib versus erlotinib or gefitinib in patients with EGFR-mutated advanced non-small-cell lung cancer. Eur J Cancer 125:49–57 31838405 10.1016/j.ejca.2019.11.006

[43] Moy B, Oliveira M, Saura C et al (2021) Neratinib+ capecitabine sustains health-related quality of life in patients with HER2-positive metastatic breast cancer and≥ 2 prior HER2-directed regimens. Breast Cancer Res Treat 188(2):449–458 33909203 10.1007/s10549-021-06217-4 PMC8260518

[44] Roy S, Grimes S, Morgan SC et al (2021) Patient-reported outcomes from a phase 3 randomized controlled trial exploring optimal sequencing of short-term androgen deprivation therapy with prostate radiation therapy in localized prostate cancer. Int J Radiat Oncol Biol Phys 110(4):1101–1113 33524545 10.1016/j.ijrobp.2021.01.032

[45] Vogel A, Qin S, Kudo M et al (2021) Lenvatinib versus sorafenib for first-line treatment of unresectable hepatocellular carcinoma: patient-reported outcomes from a randomised, open-label, non-inferiority, phase 3 trial. Lancet Gastroenterol Hepatol 6(8):649–658 34087115 10.1016/S2468-1253(21)00110-2

[46] Majem M, Goldman JW, John T et al (2022) Health-related quality of life outcomes in patients with resected epidermal growth factor receptor–mutated non–small cell lung cancer who received adjuvant osimertinib in the Phase III ADAURA trial. Clin Cancer Res 28(11):2286–2296 35012927 10.1158/1078-0432.CCR-21-3530 PMC9359973

[47] Tombal B, Saad F, Penson D et al (2019) Patient-reported outcomes following enzalutamide or placebo in men with non-metastatic, castration-resistant prostate cancer (PROSPER): a multicentre, randomised, double-blind, phase 3 trial. Lancet Oncol 20(4):556–569 30770294 10.1016/S1470-2045(18)30898-2

[48] Grivas P, Kopyltsov E, Su P-J et al (2022) Patient-reported outcomes from JAVELIN Bladder 100: avelumab first-line maintenance plus best supportive care versus best supportive care alone for advanced urothelial carcinoma. Eur Urol 83(4):320–328 35654659 10.1016/j.eururo.2022.04.016

[49] Kaufman PA, Toi M, Neven P et al (2020) Health-related quality of life in MONARCH 2: abemaciclib plus fulvestrant in hormone receptor-positive, HER2-negative advanced breast cancer after endocrine therapy. Oncologist 25(2):e243–e251 32043763 10.1634/theoncologist.2019-0551 PMC7011625

[50] Yamaguchi K, Shimada Y, Hironaka S et al (2021) Quality of life associated with ramucirumab treatment in patients with advanced gastric cancer in Japan: exploratory analysis from the Phase III RAINBOW trial. Clin Drug Invest 41(1):53–64 10.1007/s40261-020-00979-3 PMC7815617 33355909

[51] Yoh K, Atagi S, Reck M et al (2020) Patient-reported outcomes in RELAY, a phase 3 trial of ramucirumab plus erlotinib versus placebo plus erlotinib in untreated EGFR-mutated metastatic non-small-cell lung cancer. Curr Med Res Opin 36(10):1667–1675 32780643 10.1080/03007995.2020.1808781

[52] Li J, Cheng Y, Bai C et al (2022) Health-related quality of life in patients with advanced well-differentiated pancreatic and extrapancreatic neuroendocrine tumors treated with surufatinib versus placebo: results from two randomized, double-blind, phase III trials (SANET-p and SANET-ep). Eur J Cancer 169:1–9 35489301 10.1016/j.ejca.2022.03.027

[53] Harrington KJ, Ferris RL, Blumenschein Jr G et al (2017) Nivolumab versus standard, single-agent therapy of investigator’s choice in recurrent or metastatic squamous cell carcinoma of the head and neck (CheckMate 141): health-related quality-of-life results from a randomised, phase 3 trial. Lancet Oncol 18(8):1104–1115 28651929 10.1016/S1470-2045(17)30421-7 PMC6461049

[54] Witjes JA, Galsky MD, Gschwend JE et al (2022) Health-related quality of life with adjuvant nivolumab after radical resection for high-risk muscle-invasive urothelial carcinoma: results from the phase 3 CheckMate 274 trial. Eur Urol Oncol 5(5):553–563 35288066 10.1016/j.euo.2022.02.003 PMC10062393

[55] Breslow N (1974) Covariance analysis of censored survival data. Biometrics 30(1):89–99 4813387

[56] Calvert M, King M, Mercieca-Bebber R et al (2021) SPIRIT-PRO extension explanation and elaboration: guidelines for inclusion of patient-reported outcomes in protocols of clinical trials. BMJ Open 11(6):e045105. 10.1136/bmjopen-2020-045105 34193486 10.1136/bmjopen-2020-045105 PMC8246371

[57] Lawrance R, Degtyarev E, Griffiths P et al (2020) What is an estimand & how does it relate to quantifying the effect of treatment on patient-reported quality of life outcomes in clinical trials? J Patient-rep Outcomes 4(1):1–8 10.1186/s41687-020-00218-5 PMC7445213 32833083

[58] Calvert M, Blazeby J, Altman DG et al (2013) Reporting of patient-reported outcomes in randomized trials: the CONSORT PRO extension. JAMA 309(8):814–822. 10.1001/jama.2013.879 23443445 10.1001/jama.2013.879

[59] International Council for Harmonisation (ICH) (2020) Addendum on estimands and sensitivity analysis in clinical trials to the guideline on statistical principles for clinical trials E9 (R1). Step 5. Fed Regist 1–19

[60] Coens C, Pe M, Dueck AC et al (2020) International standards for the analysis of quality-of-life and patient-reported outcome endpoints in cancer randomised controlled trials: recommendations of the SISAQOL Consortium. Lancet Oncol 21(2):e83–e96. 10.1016/s1470-2045(19)30790-9 32007209 10.1016/S1470-2045(19)30790-9

[61] Novartis Pharmaceuticals Corporation (2019) Zykadia® (ceritinib) [package insert] [Internet]. U.S Food and Drug Administration website

[62] European Medicines Agency (2015) Zykadia: EPAR - product information

