# Supplementary material for: Time to deterioration of patient-reported outcome endpoints in cancer clinical trials: targeted literature review and best practice recommendations
Source: J Patient Rep Outcomes. 2024 Dec 18;8:150. doi: 10.1186/s41687-024-00824-7 (PMC11655910; doi:10.1186/s41687-024-00824-7)
Supplement: Supplementary file 1 — Supplementary Material 1 [file 41687_2024_824_MOESM1_ESM.docx]

# Appendix A

| **Table A.1. OVID search terms** | | |
| --- | --- | --- |
| **Search string** | **Search terms** | **Search term types** |
| **1** | exp neoplasms/ | Subject heading^†^ |
| **2** | ((time until adj3 (worsen* or deterior*)) or (time to adj3 (worsen* or deterior*)) or tudd or ttw).mp. | Keywords |
| **3** | 1 and 2 |  |
| **4** | Limit 3 to English language |  |
| **5** | Limit 4 to 2017-2022 (current) |  |
| **6** | Exclude conference abstracts |  |
| **7** | Deduplicate |  |
| *^†^Subject heading searches included MeSH terms.*  **An asterisk denotes a search command operator used to retrieve variants of a keyword term (e.g., random AND randomized, randomization);*  *‘exp’ denotes an exploded subject heading including all narrower subject headings within its tree hierarchy; ‘.mp’ refers to multi-purpose fields (e.g., title, abstract, original title, name of substance word, Subject Heading word, keyword heading word).* | | |

| **Table A.2. Data extraction spreadsheet description** | | |
| --- | --- | --- |
| **Tab** | **Data extraction spreadsheet** | **Description/purpose** |
| **1** | **Publication information** | Contains article information (name of study, National Clinical Trial (NCT) number, first author, year, article title, journal, issue and pages, publication type and linked publications), and structured as one row per article. |
| **2** | **Study information** | Contains clinical trial information (study objectives, design, primary cancer type, cancer stage, study region, overall N randomized (ITT), treatment of standard and novel groups, N randomized to each treatment group, dose schedule, conditions for treatment cessation). |
| **3** | **Participant characteristics** | Contains clinical trial participant information (age mean, SD, median and range, N (%) of males and females, performance status and N (%) for each PS score). |
| **4** | **PRO assessment schedule** | Contains schedule of assessments, including name of PRO measure(s) used, consistency across treatment and study period, whether there was a baseline assessment, information on ‘on vs off-treatment’ assessment schedules, whether off-treatment assessments were included in TTD Endpoint, maximum number of PRO assessments, longest recorded PRO assessment (week), whether post progression assessments were collected, their frequency and whether they were included in TTD endpoint. This information will be used to consider robustness and group results (e.g., if few assessments spread over a long period of time or if schedule different across arms, then the results may be questionable). |
| **5** | **PRO TTD endpoints and analysis methods** | Contains a list of all TTD endpoints reported (which PRO measure(s) and subscales/items) and information about multiplicity procedures, endpoint status, numeric change indicating a deterioration – responder definition (RD), reference for RD, factors in statistical model, statistical analysis technique, methodology for ties and sensitivity analyses. Assigns an endpoint ID for extraction in subsequent tabs. |
| **6** | **Endpoint definition** | Contains a checklist to assign each endpoint to a definition reported in Anota et al^9^: type of deterioration, components of composite definition, deterioration as an event, death as an event, disease progression as an event, no PRO scores rule, baseline missing rule, only baseline rule and missing visits rule. |
| **7** | **PRO TTD results** | Contains data from the TTD analyses including number of events, median time unit, median TTD, Difference (B-A) in median (months), HR, lower confidence limit (LCL), upper confidence limit (UCL), p-value, KM plot and conclusion, so the feasibility of using the endpoint definition in future trials can be assessed. |
| **8** | **Primary efficacy results** | To examine the significance of TTD endpoints alongside primary efficacy results, namely efficacy endpoint, analysis methods and statistical significance. |
| **9** | **Relationship between efficacy and TTD** | To record whether articles have conducted further analysis to assess the relationship between TTD and clinical endpoints, the analysis method used and the conclusion. |

| **Table A.3. List of Randomized Controlled Trials included in review (N=70)** | | | | | | | | |
| --- | --- | --- | --- | --- | --- | --- | --- | --- |
| **Trial name** | **Registration number** | **Study design** | **Primary cancer type** | **Cancer stage** | | **Total participants randomized (ITT)** | **Number of TTD endpoints (endpoint-treatment arm comparisons)** | **Reference** |
| ABSOLUTE | JapicCTI-132059 | Open-Label | Gastric | Advanced/ metastatic | | 741 | 1 (1) | Fujitani K, Shitara K, Takashima A, Koeda K, Hara H, Nakayama N, Hironaka S, Nishikawa K, Kimura Y, Amagai K, Hosaka H. Effect of early tumor response on the health-related quality of life among patients on second-line chemotherapy for advanced gastric cancer in the ABSOLUTE trial. Gastric Cancer. 2021 Mar;24:467-76. DOI: 10.1007/s10120-020-01131-y |
| ADAURA | NCT02511106 | Double-blinded | Lung | Both | | 682 | 20 (20) | Majem M, Goldman JW, John T, Grohe C, Laktionov K, Kim SW, Kato T, Vu HV, Lu S, Li S, Lee KY. Health-Related Quality of Life Outcomes in Patients with Resected Epidermal Growth Factor Receptor–Mutated Non–Small Cell Lung Cancer Who Received Adjuvant Osimertinib in the Phase III ADAURA Trial. Clinical Cancer Research. 2022 Jun 1;28(11):2286-96. DOI: 10.1158/1078-0432.CCR-21-3530 |
| ADJUVANT | NCT01405079 | Open-Label | Lung | Both | | 222 | 3 (3) | Zeng J, Mao WM, Chen QX, Luo TB, Wu YL, Zhou Q, Yang XN, Yan HH, Zhong WZ, Wang Q, Xu ST. Quality of life with adjuvant gefitinib versus vinorelbine plus cisplatin in patients with completely resected stage II–IIIA (N1–N2) EGFR-mutant non-small-cell lung cancer: Results from the ADJUVANT (CTONG1104) study. Lung Cancer. 2020 Dec 1;150:164-71. DOI: 10.1016/j.lungcan.2020.09.027 |
| ALEX | NCT02075840 | Open-Label | Lung | Advanced/ metastatic | | 303 | 6 (6) | Pérol M, Pavlakis N, Levchenko E, Platania M, Oliveira J, Novello S, Chiari R, Moran T, Mitry E, Nüesch E, Liu T. Patient-reported outcomes from the randomized phase III ALEX study of alectinib versus crizotinib in patients with ALK-positive non-small-cell lung cancer. Lung cancer. 2019 Dec 1;138:79-87. DOI: 10.1016/j.lungcan.2019.10.002 |
| ALTA-1L | NCT02737501 | Double-blinded | Lung | Advanced/ metastatic | | 262 | 19 (19) | Campelo MR, Lin HM, Zhu Y, Pérol M, Jahanzeb M, Popat S, Zhang P, Camidge DR. Health-related quality of life in the randomized phase III trial of brigatinib vs crizotinib in advanced ALK inhibitor–naive ALK+ non− small cell lung cancer (ALTA-1L). Lung Cancer. 2021 May 1;155:68-77. DOI: 10.1016/j.lungcan.2021.03.005 |
| ARAMIS | NCT02200614 | Double-blinded | Prostate | Non-metastatic | | 1509 | 7 (7) | Smith MR, Shore N, Tammela TL, Ulys A, Vjaters E, Polyakov S, Jievaltas M, Luz M, Alekseev B, Kuss I, Le Berre MA. Darolutamide and health-related quality of life in patients with non-metastatic castration-resistant prostate cancer: An analysis of the phase III ARAMIS trial. European Journal of Cancer. 2021 Sep 1;154:138-46. DOI: 10.1016/j.ejca.2021.06.010 |
| ASPIRE | NCT01080391 | Open-Label | Multiple Myeloma | Relapsed/ refractory | | 792 | 18 (18) | Weisel K, Ludwig H, Rieth A, Lebioda A, Goldschmidt H. Health-related quality of life of carfilzomib-and daratumumab-based therapies in patients with relapsed/refractory multiple myeloma, based on German benefit assessment data. Quality of Life Research. 2020 Jan;29:69-79. DOI: 10.1007/s11136-019-02307-5 |
| AURA3 | NCT02151981 | Open-Label | Lung | Advanced/ metastatic | | 419 | 5 (10) | Lee CK, Novello S, Rydén A, Templeton A, Rüdell K, Mann H, Ghiorghiu S, Mok T. Patient-reported symptoms and impact of treatment with osimertinib versus chemotherapy in advanced non-small-cell lung cancer: the AURA3 trial. Journal of clinical oncology. 2018:1853-60. DOI: 732-183X/18/3618w-1853w/$20.00 |
| CASPIAN | NCT03043872 | Open-Label | Lung | Advanced/ metastatic | | 805 | 20 (20) | Goldman JW, Garassino MC, Chen Y, Özgüroğlu M, Dvorkin M, Trukhin D, Statsenko G, Hotta K, Ji JH, Hochmair MJ, Voitko O. Patient-reported outcomes with first-line durvalumab plus platinum-etoposide versus platinum-etoposide in extensive-stage small-cell lung cancer (CASPIAN): a randomized, controlled, open-label, phase III study. Lung Cancer. 2020 Nov 1;149:46-52. DOI: 10.1016/j.lungcan.2020.09.003 |
| CASTOR | NCT02136134 | Open-Label | Multiple Myeloma | Relapsed/ refractory | | 498 | 15 (15) | Weisel K, Ludwig H, Rieth A, Lebioda A, Goldschmidt H. Health-related quality of life of carfilzomib-and daratumumab-based therapies in patients with relapsed/refractory multiple myeloma, based on German benefit assessment data. Quality of Life Research. 2020 Jan;29:69-79. DOI: 10.1007/s11136-019-02307-5 |
| CheckMate 141 | NCT02105636 | Open-Label | Head & Neck | Advanced/ metastatic | | 361 | 36 (72) | Harrington KJ, Ferris RL, Blumenschein G, Colevas AD, Fayette J, Licitra L, Kasper S, Even C, Vokes EE, Worden F, Saba NF. Nivolumab versus standard, single-agent therapy of investigator's choice in recurrent or metastatic squamous cell carcinoma of the head and neck (CheckMate 141): health-related quality-of-life results from a randomised, phase 3 trial. The Lancet Oncology. 2017 Aug 1;18(8):1104-15. DOI: 10.1016/S1470-2045(17)30421-7 |
| CheckMate 227 | NCT02477826 | Open-Label | Lung | Advanced/ metastatic | | 1166 | 5 (5) | Reck M, Ciuleanu TE, Lee JS, Schenker M, Audigier-Valette C, Zurawski B, Linardou H, Otterson GA, Salman P, Nishio M, de la Mora Jimenez E. First-line nivolumab plus ipilimumab versus chemotherapy in advanced NSCLC with 1% or greater tumor PD-L1 expression: patient-reported outcomes from CheckMate 227 Part 1. Journal of Thoracic Oncology. 2021 Apr 1;16(4):665-76. DOI: 10.1016/j.jtho.2020.12.019 |
| CheckMate 274 | NCT02632409 | Double-blinded | Urothelial | Not reported | | 709 | 5 (5) | Witjes JA, Galsky MD, Gschwend JE, Broughton E, Braverman J, Nasroulah F, Maira-Arce M, Ye X, Shi L, Guo S, Hamilton M. Health-related quality of life with adjuvant nivolumab after radical resection for high-risk muscle-invasive urothelial carcinoma: results from the phase 3 CheckMate 274 trial. European urology oncology. 2022 Oct 1;5(5):553-63. DOI: 10.1016/j.euo.2022.02.003 |
| CheckMate 743 | NCT02899299 | Open-Label | Lung | Not reported | | 605 | 35 (35) | Scherpereel A, Antonia S, Bautista Y, Grossi F, Kowalski D, Zalcman G, Nowak AK, Fujimoto N, Peters S, Tsao AS, Mansfield AS. First-line nivolumab plus ipilimumab versus chemotherapy for the treatment of unresectable malignant pleural mesothelioma: patient-reported outcomes in CheckMate 743. Lung Cancer. 2022 May 1;167:8-16. DOI: 10.1016/j.lungcan.2022.03.012 |
| CheckMate 9ER | NCT03141177 | Open-Label | Kidney | Advanced/ metastatic | | 651 | 13 (13) | Cella D, Motzer RJ, Suarez C, Blum SI, Ejzykowicz F, Hamilton M, Wallace JF, Simsek B, Zhang J, Ivanescu C, Apolo AB. Patient-reported outcomes with first-line nivolumab plus cabozantinib versus sunitinib in patients with advanced renal cell carcinoma treated in CheckMate 9ER: an open-label, randomised, phase 3 trial. The Lancet Oncology. 2022 Feb 1;23(2):292-303. DOI: 10.1016/S1470-2045(21)00693-8 |
| CLEAR | NCT02811861 | Open-Label | Kidney | Advanced/ metastatic | | 1,069 | 5 (5) | Motzer R, Porta C, Alekseev B, Rha SY, Choueiri TK, Mendez-Vidal MJ, Hong SH, Kapoor A, Goh JC, Eto M, Bennett L. Health-related quality-of-life outcomes in patients with advanced renal cell carcinoma treated with lenvatinib plus pembrolizumab or everolimus versus sunitinib (CLEAR): a randomised, phase 3 study. The Lancet Oncology. 2022 Jun 1;23(6):768-80. DOI: 10.1016/S1470-2045(22)00212-1 |
| CONCUR | NCT01584830 | Double-blinded | Colorectal | Advanced/ metastatic | | 204 | 15 (15) | Hofheinz RD, Bruix J, Demetri GD, Grothey A, Marian M, Bartsch J, Odom D. Effect of regorafenib in delaying definitive deterioration in health-related quality of life in patients with advanced cancer of three different tumor types. Cancer Management and Research. 2021 Jul 12:5523-33. DOI: 10.2147/CMAR.S305939 |
| CORRECT | NCT01103323 | Double-blinded | Colorectal | Advanced/ metastatic | | 760 | 12 (12) | Hofheinz RD, Bruix J, Demetri GD, Grothey A, Marian M, Bartsch J, Odom D. Effect of regorafenib in delaying definitive deterioration in health-related quality of life in patients with advanced cancer of three different tumor types. Cancer Management and Research. 2021 Jul 12:5523-33. DOI: 10.2147/CMAR.S305939 |
| EF-14 | NCT00916409 | Open-Label | Glioblastoma | Not reported | | 695 | 9 (9) | Taphoorn MJ, Dirven L, Kanner AA, Lavy-Shahaf G, Weinberg U, Taillibert S, Toms SA, Honnorat J, Chen TC, Sroubek J, David C. Influence of treatment with tumor-treating fields on health-related quality of life of patients with newly diagnosed glioblastoma: a secondary analysis of a randomized clinical trial. JAMA oncology. 2018 Apr 1;4(4):495-504. DOI: 10.1001/jamaoncol.2017.5082 |
| ENDEAVOR | NCT01568866 | Open-Label | Multiple Myeloma | Relapsed/ refractory | | 2788 | 5 (5) | Weisel K, Ludwig H, Rieth A, Lebioda A, Goldschmidt H. Health-related quality of life of carfilzomib-and daratumumab-based therapies in patients with relapsed/refractory multiple myeloma, based on German benefit assessment data. Quality of Life Research. 2020 Jan;29:69-79. DOI: 10.1007/s11136-019-02307-5 |
| FLAURA | NCT02296125 | Double-blinded | Lung | Advanced/ metastatic | | 556 | 5 (5) | Leighl NB, Karaseva N, Nakagawa K, Cho BC, Gray JE, Hovey T, Walding A, Rydén A, Novello S. Patient-reported outcomes from FLAURA: osimertinib versus erlotinib or gefitinib in patients with EGFR-mutated advanced non-small-cell lung cancer. European Journal of Cancer. 2020 Jan 1;125:49-57. DOI: 10.1016/j.ejca.2019.11.006 |
| GADOLIN | NCT01059630 | Open-Label | Lymphoma | Both | | 396 | 1 (1) | Cheson BD, Trask PC, Gribben JG, Dimier N, Kimby E, Lugtenburg PJ, Thieblemont C, Wassner-Fritsch E, Launonen A, Sehn LH. Health-related quality of life and symptoms in patients with rituximab-refractory indolent non-Hodgkin lymphoma treated in the phase III GADOLIN study with obinutuzumab plus bendamustine versus bendamustine alone. Annals of hematology. 2017 Feb;96:253-9. DOI: 10.1007/s00277-016-2878-5 |
| GRID | NCT01271712 | Double-blinded | Gastrointestinal Stromal | Advanced/ metastatic | | 199 | 5 (5) | Hofheinz RD, Bruix J, Demetri GD, Grothey A, Marian M, Bartsch J, Odom D. Effect of regorafenib in delaying definitive deterioration in health-related quality of life in patients with advanced cancer of three different tumor types. Cancer Management and Research. 2021 Jul 12:5523-33. DOI: 10.2147/CMAR.S305939 |
| IMbrave 150 | NCT03434379 | Open-Label | Liver | Advanced/ metastatic | | 501 | 23 (23) | Galle PR, Finn RS, Qin S, Ikeda M, Zhu AX, Kim TY, Kudo M, Breder V, Merle P, Kaseb A, Li D. Patient-reported outcomes with atezolizumab plus bevacizumab versus sorafenib in patients with unresectable hepatocellular carcinoma (IMbrave150): an open-label, randomised, phase 3 trial. The Lancet Oncology. 2021 Jul 1;22(7):991-1001. DOI: 10.1016/S1470-2045(21)00151-0 |
| IMmotion 151 | NCT02420821 | Open-Label | Kidney | Advanced/ metastatic | | 915 | 6 (6) | Atkins MB, Rini BI, Motzer RJ, Powles T, McDermott DF, Suarez C, Bracarda S, Stadler WM, Donskov F, Gurney H, Oudard S. Patient-reported outcomes from the phase III Randomized IMmotion151 Trial: Atezolizumab+ Bevacizumab versus sunitinib in treatment-naive metastatic renal cell carcinoma. Clinical Cancer Research. 2020 Jun 1;26(11):2506-14. DOI: 10.1158/1078-0432.CCR-19-2838 |
| IMpassion 130 | NCT02425891 | Double-blinded | Breast | Advanced/ metastatic | | 902 | 4 (4) | Adams S, Diéras V, Barrios CH, Winer EP, Schneeweiss A, Iwata H, Loi S, Patel S, Henschel V, Chui SY, Rugo HS. Patient-reported outcomes from the phase III IMpassion130 trial of atezolizumab plus nab-paclitaxel in metastatic triple-negative breast cancer. Annals of Oncology. 2020 May 1;31(5):582-9. DOI: 10.1016/j.annonc.2020.02.003 |
| JAVELIN Bladder 100 | NCT02603432 | Open-Label | Urothelial | Advanced/ metastatic | | 700 | 6 (6) | Grivas P, Kopyltsov E, Su PJ, Parnis FX, Park SH, Yamamoto Y, Fong PC, Tournigand C, Duran MA, Bamias A, Caserta C. Patient-reported outcomes from JAVELIN Bladder 100: avelumab first-line maintenance plus best supportive care versus best supportive care alone for advanced urothelial carcinoma. European Urology. 2023 Apr 1;83(4):320-8. DOI: 10.1016/j.eururo.2022.04.016 |
| KEYNOTE-024 | NCT02142738 | Open-Label | Lung | Advanced/ metastatic | | 305 | 1 (1) | Brahmer JR, Rodríguez-Abreu D, Robinson AG, Hui R, Csőszi T, Fülöp A, Gottfried M, Peled N, Tafreshi A, Cuffe S, O'Brien M. Health-related quality-of-life results for pembrolizumab versus chemotherapy in advanced, PD-L1-positive NSCLC (KEYNOTE-024): a multicentre, international, randomised, open-label phase 3 trial. The Lancet Oncology. 2017 Dec 1;18(12):1600-9. DOI: |
| KEYNOTE-040 | NCT02252042 | Open-Label | Head & Neck | Advanced/ metastatic | | 495 | 35 (35) | Harrington KJ, Soulières D, Le Tourneau C, Dinis J, Licitra LF, Ahn MJ, Soria A, Machiels JP, Mach N, Mehra R, Burtness B. Quality of life with pembrolizumab for recurrent and/or metastatic head and neck squamous cell carcinoma: KEYNOTE-040. JNCI: Journal of the National Cancer Institute. 2021 Feb 1;113(2):171-81. DOI: 10.1093/jnci/djaa063 |
| KEYNOTE-045 | NCT02256436 | Open-Label | Urothelial | Advanced/ metastatic | | 542 | 2 (2) | Vaughn DJ, Bellmunt J, Fradet Y, Lee JL, Fong L, Vogelzang NJ, Climent MA, Petrylak DP, Choueiri TK, Necchi A, Gerritsen W. Health-related quality-of-life analysis from KEYNOTE-045: a phase III study of pembrolizumab versus chemotherapy for previously treated advanced urothelial cancer. Journal of Clinical Oncology. 2018 Jun 1;36(16):1579-87. 10.1200/JCO.2017.76.9562 |
| KEYNOTE-048 | NCT02358031 | Open-Label | Head & Neck | Advanced/ metastatic | | 882 | 3 (6) | Rischin D, Harrington KJ, Greil R, Soulieres D, Tahara M, de Castro Jr G, Psyrri A, Brana I, Neupane P, Bratland Å, Fuereder T. Pembrolizumab alone or with chemotherapy for recurrent or metastatic head and neck squamous cell carcinoma: Health-related quality-of-life results from KEYNOTE-048. Oral oncology. 2022 May 1;128:105815. DOI: 10.1016/j.oraloncology.2022.105815 |
| KEYNOTE-062 | NCT02494583 | Partial-blind | Esophageal | Advanced/ metastatic | | 763 | 4 (4) | Van Cutsem E, Valderrama A, Bang YJ, Fuchs CS, Shitara K, Janjigian YY, Qin S, Larson TG, Shankaran V, Stein S, Norquist JM. Quality of life with first-line pembrolizumab for PD-L1–positive advanced gastric/gastroesophageal junction adenocarcinoma: results from the randomised phase III KEYNOTE-062 study. ESMO open. 2021 Aug 1;6(4):100189. DOI: 10.1016/j.esmoop.2021.100189 |
| KEYNOTE-177 | NCT02563002 | Open-Label | Colorectal | Advanced/ metastatic | | 307 | 5 (5) | Andre T, Amonkar M, Norquist JM, Shiu KK, Kim TW, Jensen BV, Jensen LH, Punt CJ, Smith D, Garcia-Carbonero R, Sevilla I. Health-related quality of life in patients with microsatellite instability-high or mismatch repair deficient metastatic colorectal cancer treated with first-line pembrolizumab versus chemotherapy (KEYNOTE-177): an open-label, randomised, phase 3 trial. The Lancet Oncology. 2021 May 1;22(5):665-77. DOI: 10.1016/S1470-2045(21)00064-4 |
| KEYNOTE-181 | NCT02564263 | Open-Label | Esophageal | Advanced/ metastatic | | 628 | 3 (3) | Adenis A, Kulkarni AS, Girotto GC, de la Fouchardiere C, Senellart H, van Laarhoven HW, Mansoor W, Al-Rajabi R, Norquist J, Amonkar M, Suryawanshi S. Impact of pembrolizumab versus chemotherapy as second-line therapy for advanced esophageal cancer on health-related quality of life in KEYNOTE-181. Journal of Clinical Oncology. 2022 Feb 1;40(4):382-91. DOI: 10.1200/JCO.21.00601 |
| KEYNOTE-189 | NCT02578680 | Double-blinded | Lung | Advanced/ metastatic | | 616 | 1 (1) | Garassino MC, Gadgeel S, Esteban E, Felip E, Speranza G, Domine M, Hochmair MJ, Powell S, Cheng SY, Bischoff HG, Peled N. Patient-reported outcomes following pembrolizumab or placebo plus pemetrexed and platinum in patients with previously untreated, metastatic, non-squamous non-small-cell lung cancer (KEYNOTE-189): a multicentre, double-blind, randomised, placebo-controlled, phase 3 trial. The Lancet Oncology. 2020 Mar 1;21(3):387-97. DOI: 10.1016/S1470-2045(19)30801-0 |
| KEYNOTE-204 | NCT02684292 | Open-Label | Lymphoma | Not reported | | 304 | 6 (6) | Zinzani PL, Ramchandren R, Santoro A, Paszkiewicz-Kozik E, Gasiorowski R, Johnson NA, de Oliveira JS, Buccheri V, Perini GF, Dickinson M, McDonald A. Quality-of-life analysis of pembrolizumab vs brentuximab vedotin for relapsed/refractory classical Hodgkin lymphoma. Blood Advances. 2022 Jan 25;6(2):590-9. DOI: 10.1182/bloodadvances.2021004970 |
| KEYNOTE-407 | NCT02775435 | Double-blinded | Lung | Advanced/ metastatic | | 559 | 1 (1) | Mazieres J, Kowalski D, Luft A, Vicente D, Tafreshi A, Gümüş M, Laktionov K, Hermes B, Cicin I, Rodríguez-Cid J, Wilson J. Health-related quality of life with carboplatin-paclitaxel or nab-paclitaxel with or without pembrolizumab in patients with metastatic squamous non-small-cell lung cancer. Journal of Clinical Oncology. 2019. DOI: 10.1200/JCO.19.01348 |
| KEYNOTE-426 | NCT02853331 | Open-Label | Kidney | Advanced/ metastatic | | 861 | 6 (6) | Bedke J, Rini BI, Plimack ER, Stus V, Gafanov R, Waddell T, Nosov D, Pouliot F, Soulières D, Melichar B, Vynnychenko I. Health-related quality of life analysis from KEYNOTE-426: pembrolizumab plus axitinib versus sunitinib for advanced renal cell carcinoma. European Urology. 2022 Oct 1;82(4):427-39. DOI: 10.1016/j.eururo.2022.06.009 |
| LATITUDE | NCT01715285 | Double-blinded | Prostate | Advanced/ metastatic | | 1199 | 15 (15) | Chi KN, Protheroe A, Rodríguez-Antolín A, Facchini G, Suttman H, Matsubara N, Ye Z, Keam B, Damião R, Li T, McQuarrie K. Patient-reported outcomes following abiraterone acetate plus prednisone added to androgen deprivation therapy in patients with newly diagnosed metastatic castration-naive prostate cancer (LATITUDE): an international, randomised phase 3 trial. The Lancet Oncology. 2018 Feb 1;19(2):194-206. DOI: 10.1016/S1470-2045(17)30911-7 |
| MAVORIC | NCT01728805 | Open-Label | Lymphoma | Both | | 372 | 4 (4) | Porcu P, Hudgens S, Horwitz S, Quaglino P, Cowan R, Geskin L, Beylot-Barry M, Floden L, Bagot M, Tsianakas A, Moskowitz A. Quality of life effect of the anti-CCR4 monoclonal antibody mogamulizumab versus vorinostat in patients with cutaneous T-cell lymphoma. Clinical Lymphoma Myeloma and Leukemia. 2021 Feb 1;21(2):97-105. DOI: 10.1016/j.clml.2020.09.003 |
| MILES-3/MILES-4 | MILES-3: NCT01405586 MILES-4: NCT01656551 | Open-Label | Lung | Advanced/ metastatic | | 531 | 24 (24) | Morabito A, Piccirillo MC, Maione P, Luciani A, Cavanna L, Bonanno L, Filipazzi V, Leo S, Cinieri S, Morgillo F, Burgio MA. Effect on quality of life of cisplatin added to single-agent chemotherapy as first-line treatment for elderly patients with advanced non-small cell lung cancer: Joint analysis of MILES-3 and MILES-4 randomised phase 3 trials. Lung Cancer. 2019 Jul 1;133:62-8. DOI: 10.1016/j.lungcan.2019.05.009 |
| MONALEESA 3 | NCT02422615 | Double-blinded | Breast | Advanced/ metastatic | | 726 | 4 (4) | Fasching PA, Beck JT, Chan A, De Laurentiis M, Esteva FJ, Jerusalem G, Neven P, Pivot X, Bianchi GV, Martin M, Chandiwana D. Ribociclib plus fulvestrant for advanced breast cancer: Health-related quality-of-life analyses from the MONALEESA-3 study. The Breast. 2020 Dec 1;54:148-54. DOI: 10.1016/j.breast.2020.09.008 |
| MONALEESA 7 | NCT02278120 | Double-blinded | Breast | Advanced/ metastatic | | 672 | 7 (7) | Harbeck N, Franke F, Villanueva-Vazquez R, Lu YS, Tripathy D, Chow L, Babu GK, Im YH, Chandiwana D, Gaur A, Lanoue B. Health-related quality of life in premenopausal women with hormone-receptor-positive, HER2-negative advanced breast cancer treated with ribociclib plus endocrine therapy: results from a phase III randomized clinical trial (MONALEESA-7). Therapeutic Advances in Medical Oncology. 2020 Jul;12:1758835920943065. DOI: 10.1177/1758835920943065 |
| MONARCH 2 | NCT02107703 | Double-blinded | Breast | Advanced/ metastatic | | 669 | 24 (24) | Kaufman PA, Toi M, Neven P, Sohn J, Grischke EM, Andre V, Stoffregen C, Shekarriz S, Price GL, Carter GC, Sledge Jr GW. Health‐related quality of life in MONARCH 2: Abemaciclib plus fulvestrant in hormone receptor‐positive, HER2‐negative advanced breast cancer after endocrine therapy. The oncologist. 2020 Feb 1;25(2):e243-51. DOI: 10.1634/theoncologist.2019-0551 |
| MYSTIC | NCT02453282 | Open-Label | Lung | Advanced/ metastatic | | 1118 | 20 (40) | Garon EB, Cho BC, Reinmuth N, Lee KH, Luft A, Ahn MJ, Robinet G, Le Moulec S, Natale R, Schneider J, Shepherd FA. Patient-reported outcomes with durvalumab with or without tremelimumab versus standard chemotherapy as first-line treatment of metastatic non–small-cell lung cancer (MYSTIC). Clinical Lung Cancer. 2021 Jul 1;22(4):301-12. DOI: 10.1016/j.cllc.2021.02.010 |
| NALA | NCT01808573 | Open-Label | Breast | Advanced/ metastatic | | 621 | 7 (7) | Moy B, Oliveira M, Saura C, Gradishar W, Kim SB, Brufsky A, Hurvitz SA, Ryvo L, Fagnani D, Kalmadi S, Silverman P. Neratinib+ capecitabine sustains health-related quality of life in patients with HER2-positive metastatic breast cancer and ≥2 prior HER2-directed regimens. Breast cancer research and treatment. 2021 Jul;188(2):449-58. DOI: 10.1007/s10549-021-06217-4 |
| NETTER-1 | NCT01578239 | Open-Label | Midgut Neuroendocrine | Advanced/ metastatic | | 231 | 72 (72) | Strosberg J, Wolin E, Chasen B, Kulke M, Bushnell D, Caplin M, Baum RP, Kunz P, Hobday T, Hendifar A, Oberg K. Health-related quality of life in patients with progressive midgut neuroendocrine tumors treated with 177Lu-dotatate in the phase III NETTER-1 trial. Journal of Clinical Oncology. 2018 Sep 9;36(25):2578. DOI: 10.1200/JCO.2018.78.5865 |
| NRG/RTOG 0825 | NCT00884741 | Double-blinded | Glioblastoma | Non-metastatic | | 508 | 8 (8) | Wefel JS, Armstrong TS, Pugh SL, Gilbert MR, Wendland MM, Brachman DG, Roof KS, Brown PD, Crocker IR, Robins HI, Hunter G. Neurocognitive, symptom, and health-related quality of life outcomes of a randomized trial of bevacizumab for newly diagnosed glioblastoma (NRG/RTOG 0825). Neuro-oncology. 2021 Jul 1;23(7):1125-38. DOI: 10.1093/neuonc/noab011 |
| OAK | NCT02008227 | Open-Label | Lung | Advanced/ metastatic | | 850 | 6 (6) | Bordoni R, Ciardiello F, von Pawel J, Cortinovis D, Karagiannis T, Ballinger M, Sandler A, Yu W, He P, Matheny C, Felizzi F. Patient-reported outcomes in OAK: a phase III study of atezolizumab versus docetaxel in advanced non–small-cell lung cancer. Clinical Lung Cancer. 2018 Sep 1;19(5):441-9. DOI: 10.1016/j.cllc.2018.05.011 |
| OlympiAD | NCT02000622 | Open-Label | Breast | Advanced/ metastatic | | 302 | 9 (9) | Robson M, Ruddy KJ, Seock-Ah IM, Senkus E, Xu B, Domchek SM, Masuda N, Li W, Tung N, Armstrong A, Delaloge S. Patient-reported outcomes in patients with a germline BRCA mutation and HER2-negative metastatic breast cancer receiving olaparib versus chemotherapy in the OlympiAD trial. European Journal of Cancer. 2019 Oct 1;120:20-30. DOI: 10.1016/j.ejca.2019.06.023 |
| OTT 0101 | Not reported | Open-Label | Prostate | Both | | 393 | 15 (15) | Roy S, Grimes S, Morgan SC, Eapen L, Malone J, Craig J, Spratt DE, Malone S. Patient-reported outcomes from a phase 3 randomized controlled trial exploring optimal sequencing of short-term androgen deprivation therapy with prostate radiation therapy in localized prostate cancer. International Journal of Radiation Oncology* Biology* Physics. 2021 Jul 15;110(4):1101-13. DOI: 10.1016/j.ijrobp.2021.01.032 |
| PACIFIC | NCT02125461 | Double-blinded | Lung | Both | | 713 | 40 (40) | Hui R, Özgüroğlu M, Villegas A, Daniel D, Vicente D, Murakami S, Yokoi T, Chiappori A, Lee KH, de Wit M, Cho BC. Patient-reported outcomes with durvalumab after chemoradiotherapy in stage III, unresectable non-small-cell lung cancer (PACIFIC): a randomised, controlled, phase 3 study. The Lancet Oncology. 2019 Dec 1;20(12):1670-80. DOI: 10.1016/S1470-2045(19)30519-4 |
| PEARL | NCT02028507 | Open-Label | Breast | Advanced/ metastatic | | 601 | 25 (25) | Kahan Z, Gil-Gil M, Ruiz-Borrego M, Carrasco E, Ciruelos E, Muñoz M, Bermejo B, Margeli M, Antón A, Casas M, Csöszi T. Health-related quality of life with palbociclib plus endocrine therapy versus capecitabine in postmenopausal patients with hormone receptor–positive metastatic breast cancer: Patient-reported outcomes in the PEARL study. European Journal of Cancer. 2021 Oct 1;156:70-82. DOI: 10.1016/j.ejca.2021.07.004 |
| POLLUX | NCT02076009 | Open-Label | Multiple Myeloma | Relapsed/ refractory | | 569 | 15 (15) | Weisel K, Ludwig H, Rieth A, Lebioda A, Goldschmidt H. Health-related quality of life of carfilzomib-and daratumumab-based therapies in patients with relapsed/refractory multiple myeloma, based on German benefit assessment data. Quality of Life Research. 2020 Jan;29:69-79. DOI: 10.1007/s11136-019-02307-5 |
| PRODIGE 12 | NCT01313377 | Open-Label | Biliary Tract | Both | | 194 | 3 (3) | Edeline J, Benabdelghani M, Bertaut A, Watelet J, Hammel P, Joly JP, Boudjema K, Fartoux L, Bouhier-Leporrier K, Jouve JL, Faroux R. Gemcitabine and oxaliplatin chemotherapy or surveillance in resected biliary tract cancer (PRODIGE 12-ACCORD 18-UNICANCER GI): a randomized phase III study. Journal of Clinical Oncology. 2019 Mar 10;37(8):658-67. DOI: 10.1200/JCO.18.00050 |
| PROFILE 1029 | NCT01639001 | Open-Label | Lung | Advanced/ metastatic | | 207 | 1 (1) | Wu YL, Lu S, Lu Y, Zhou J, Shi YK, Sriuranpong V, Ho JC, Ong CK, Tsai CM, Chung CH, Wilner KD. Results of PROFILE 1029, a phase III comparison of first-line crizotinib versus chemotherapy in East Asian patients with ALK-positive advanced non–small cell lung cancer. Journal of Thoracic Oncology. 2018 Oct 1;13(10):1539-48. DOI: 10.1016/j.jtho.2018.06.012 |
| PROMID | NCT00171873 | Double-blinded | Midgut Neuroendocrine | Advanced/ metastatic | | 85 | 18 (18) | Rinke A, Neary MP, Eriksson J, Hunger M, Doan T, Karli D, Arnold R. Health-related quality of life for long-acting octreotide versus placebo in patients with metastatic midgut neuroendocrine tumors in the phase 3 PROMID trial. Neuroendocrinology. 2019 Aug 20;109(2):141-51. DOI: 10.1159/000499469 |
| PROSPER | NCT02003924 | Double-blinded | Prostate | Non-metastatic | | 1401 | 28 (28) | Tombal B, Saad F, Penson D, Hussain M, Sternberg CN, Morlock R, Ramaswamy K, Ivanescu C, Attard G. Patient-reported outcomes following enzalutamide or placebo in men with non-metastatic, castration-resistant prostate cancer (PROSPER): a multicentre, randomised, double-blind, phase 3 trial. The Lancet Oncology. 2019 Apr 1;20(4):556-69. DOI: 10.1016/S1470-2045(18)30898-2 |
| RADIANT 4 | NCT01524783 | Double-blinded | Neuroendocrine | Non-metastatic | | 302 | 5 (5) | Pavel ME, Singh S, Strosberg JR, Bubuteishvili-Pacaud L, Degtyarev E, Neary MP, Carnaghi C, Tomasek J, Wolin E, Raderer M, Lahner H. Health-related quality of life for everolimus versus placebo in patients with advanced, non-functional, well-differentiated gastrointestinal or lung neuroendocrine tumours (RADIANT-4): a multicentre, randomised, double-blind, placebo-controlled, phase 3 trial. The lancet oncology. 2017 Oct 1;18(10):1411-22. DOI: 10.1016/S1470-2045(17)30471-0 |
| RAINBOW | NCT01170663 | Double-blinded | Gastric | Advanced/ metastatic | | 140 | 15 (15) | Yamaguchi K, Shimada Y, Hironaka S, Sugimoto N, Komatsu Y, Nishina T, Omuro Y, Tamura T, Piao Y, Homma G, Jen MH. Quality of Life Associated with Ramucirumab Treatment in Patients with Advanced Gastric Cancer in Japan: Exploratory Analysis from the Phase III RAINBOW Trial. Clinical Drug Investigation. 2021 Jan;41:53-64. DOI: 10.1007/s40261-020-00979-3 |
| RATIONALE 302 | NCT03430843 | Open-Label | Esophageal | Advanced/ metastatic | | 512 | 6 (6) | Van Cutsem E, Kato K, Ajani J, Shen L, Xia T, Ding N, Zhan L, Barnes G, Kim SB. Tislelizumab versus chemotherapy as second-line treatment of advanced or metastatic esophageal squamous cell carcinoma (RATIONALE 302): impact on health-related quality of life. ESMO open. 2022 Aug 1;7(4):100517. DOI: 10.1016/j.esmoop.2022.100517 |
| REACH 2 | NCT02435433 | Double-blinded | Liver | Advanced/ metastatic | | 542 | 11 (11) | Zhu AX, Nipp RD, Finn RS, Galle PR, Llovet JM, Blanc JF, Okusaka T, Chau I, Cella D, Girvan A, Gable J. Ramucirumab in the second-line for patients with hepatocellular carcinoma and elevated alpha-fetoprotein: patient-reported outcomes across two randomised clinical trials. ESMO open. 2020 Jan 1;5(4):e000797. DOI: 10.1136/esmoopen-2020-000797 |
| REFLECT | NCT01761266 | Open-Label | Liver | Advanced/ metastatic | | 931 | 25 (25) | Vogel A, Qin S, Kudo M, Su Y, Hudgens S, Yamashita T, Yoon JH, Fartoux L, Simon K, López C, Sung M. Lenvatinib versus sorafenib for first-line treatment of unresectable hepatocellular carcinoma: patient-reported outcomes from a randomised, open-label, non-inferiority, phase 3 trial. The Lancet Gastroenterology & Hepatology. 2021 Aug 1;6(8):649-58. DOI: 10.1016/S2468-1253(21)00110-2 |
| RELAY | NCT02411448 | Double-blinded | Lung | Advanced/ metastatic | | 449 | 11 (11) | Yoh K, Atagi S, Reck M, Garon EB, Ponce Aix S, Moro-Sibilot D, Winfree KB, Frimodt-Moller B, Zimmermann A, Visseren-Grul C, Nakagawa K. Patient-reported outcomes in RELAY, a phase 3 trial of ramucirumab plus erlotinib versus placebo plus erlotinib in untreated EGFR-mutated metastatic non-small-cell lung cancer. Current Medical Research and Opinion. 2020 Oct 2;36(10):1667-75. DOI: 10.1080/03007995.2020.1808781 |
| RESORCE | NCT01774344 | Double-blinded | Liver | Advanced/ metastatic | | 573 | 2 (2) | Hofheinz RD, Bruix J, Demetri GD, Grothey A, Marian M, Bartsch J, Odom D. Effect of regorafenib in delaying definitive deterioration in health-related quality of life in patients with advanced cancer of three different tumor types. Cancer Management and Research. 2021 Jul 12:5523-33. DOI: 10.2147/CMAR.S305939 |
| SANET-p/SANET-ep | NCT02589821 | Double-blinded | Neuroendocrine | Advanced/ metastatic | | 370 | 15 (15) | Li J, Cheng Y, Bai C, Xu J, Shen L, Li J, Zhou Z, Li Z, Chi Y, Yu X, Li E. Health-related quality of life in patients with advanced well-differentiated pancreatic and extrapancreatic neuroendocrine tumors treated with surufatinib versus placebo: Results from two randomized, double-blind, phase III trials (SANET-p and SANET-ep). European Journal of Cancer. 2022 Jul 1;169:1-9. DOI: 10.1016/j.ejca.2022.03.027 |
| SEAL | NCT02588170 | Double-blinded | Liposarcoma | Advanced/ metastatic | | 277 | 4 (4) | Gounder M, Abdul Razak AR, Gilligan AM, Leong H, Ma X, Somaiah N, Chawla SP, Martin-Broto J, Grignani G, Schuetze SM, Vincenzi B. Health-related quality of life and pain with selinexor in patients with advanced dedifferentiated liposarcoma. Future Oncology. 2021 Apr;17(22):2923-39. DOI: 10.2217/fon-2021-0284 |
| SOLAR 1 | NCT02606461 | Double-blinded | Breast | Advanced/ metastatic | | 341 | 7 (7) | Ciruelos EM, Rugo HS, Mayer IA, Levy C, Forget F, Mingorance JI, Safra T, Masuda N, Park YH, Juric D, Conte P. Patient-reported outcomes in patients with PIK3CA-mutated hormone receptor–positive, human epidermal growth factor receptor 2–negative advanced breast cancer from SOLAR-1. Journal of Clinical Oncology. 2021 Jun 6;39(18):2005. DOI: 10.1200/JCO.20.0113 |
| TITAN | NCT02437318 | Double-blinded | Prostate | Advanced/ metastatic | | 1052 | 17 (17) | Agarwal N, McQuarrie K, Bjartell A, Chowdhury S, de Santana Gomes AJ, Chung BH, Özgüroğlu M, Soto ÁJ, Merseburger AS, Uemura H, Ye D. Health-related quality of life after apalutamide treatment in patients with metastatic castration-sensitive prostate cancer (TITAN): a randomised, placebo-controlled, phase 3 study. The Lancet Oncology. 2019 Nov 1;20(11):1518-30. 10.1016/S1470-2045(19)30620-5 |
| TOWER | NCT02489318 | Open-Label | Leukemia | Relapsed/ refractory | | 342 | 15 (15) | Topp MS, Zimmerman Z, Cannell P, Dombret H, Maertens J, Stein A, Franklin J, Tran Q, Cong Z, Schuh AC. Health-related quality of life in adults with relapsed/refractory acute lymphoblastic leukemia treated with blinatumomab. Blood, The Journal of the American Society of Hematology. 2018 Jun 28;131(26):2906-14. DOI: 10.1182/blood-2017-09-804658 |
|  | | | | | *ITT: Intention to treat.* | | | |

| **Table A.4. Dose Schedule and PRO assessment frequency** | | | | | | | |
| --- | --- | --- | --- | --- | --- | --- | --- |
| **Initial frequency of on-treatment PRO Assessment used for TTD analysis** | **Dose Schedule Number of trials (% of 70 trials)** | | | | | | |
|  | **Daily** | **Every week** | **Every 2 weeks** | **Every 3 weeks** | **Every 4 weeks** | **Every 6 weeks** | **Total^1^** |
| **Every Week** | 2 (2.9%) | 0 (0%) | 0 (0%) | 2 (2.9%) | 0 (0%) | 0 (0%) | 4 (5.7%) |
| **Every 2 weeks** | 0 (0%) | 0 (0%) | 2 (2.9%) | 0 (0%) | 1 (1.4%) | 0 (0%) | 3 (4.3%) |
| **Every 3 weeks** | 0 (0%) | 2 (2.9%) | 0 (0%) | 16 (22.9%) | 0 (0%) | 0 (0%) | 18 (25.7%) |
| **Every 4 weeks** | 2 (2.9%) | 0 (0%) | 4 (5.7%) | 0 (0%) | 11 (15.7%) | 0 (0%) | 17 (24.3%) |
| **Every 6 weeks** | 1 (1.4%) | 0 (0%) | 2 (2.9%) | 4 (5.7%) | 1 (1.4%) | 2 (2.9%) | 10 (14.3%) |
| **Every 8 weeks/Bi-Monthly** | 2 (2.9%) | 0 (0%) | 1 (1.4%) | 1 (1.4%) | 6 (8.6%) | 0 (0%) | 10 (14.3%) |
| **Every 12 weeks/3 months** | 1 (1.4%) | 0 (0%) | 2 (2.9%) | 0 (0%) | 3 (4.3%) | 0 (0%) | 6 (8.6%) |
| **Every 16 weeks** | 2 (2.9%) | 0 (0%) | 0 (0%) | 0 (0%) | 0 (0%) | 0 (0%) | 2 (2.9%) |
| **Varied between treatment arms** | 0 (0%) | 0 (0%) | 2 (2.9%) | 0 (0%) | 0 (0%) | 1 (1.4%) | 3 (4%) |
| **Not reported** | 0 (0%) | 0 (0%) | 0 (0%) | 2 (2.9%) | 0 (0%) | 0 (0%) | 2 (2.9%) |
| **Total^1^** | 8 (11.4%) | 2 (2.9%) | 13 (18.6%) | 23 (32.9%) | 21 (30.0%) | 3 (4.3%) |  |

*^1^ Some trials have differing assessment schedules for different PROs; hence the row and column totals may exceed the number of reviewed trials.*

| Table A.5. Broad TTD definitions by primary cancer types and stages | | | |
| --- | --- | --- | --- |
| **Primary cancer type  (n trials)** | **Broad TTD Definition assessed**  **Number of trials (% of 70 trials)** | | |
|  | **Time to first deterioration (TTFD)** | **Time to confirmed deterioration (TTCD)** | **Time until definitive deterioration (TUDD)** |
| **Cancer type** | | | |
| Lung (n=18) | 10 (14.3%) | 9 (12.9%) | 1 (1.4%) |
| Breast (n=8) | 4 (5.7%) | 1 (1.4%) | 4 (5.7%) |
| Prostate (n=5) | 3 (4.3%) | 3 (4.3%) | 1 (1.4%) |
| Kidney (n=4) | 3 (4.3%) | 2 (2.9%) | 1 (1.4%) |
| Liver (n=4) | 1 (1.4%) | 1 (1.4%) | 2 (2.9%) |
| Multiple Myeloma (n=4) | 4 (5.7%) | - | - |
| Neuroendocrine (n=4) | 1 (1.4%) | - | 4 (5.7%) |
| Colorectal (n=3) | - | 1 (1.4%) | 2 (2.9%) |
| Esophageal (n=3) | 2 (2.9%) | 1 (1.4%) | - |
| Gastric / gastrointestinal stromal (n=3) | 2 (2.9%) | - | 1 (1.4%) |
| Head & Neck (n=3) | 2 (2.9%) | 1 (1.4%) | - |
| Lymphoma (n=3) | 3 (4.3%) | - | - |
| Urothelial (n=3) | 1 (1.4%) | 2 (2.9%) | - |
| Glioblastoma (n=2) | 1 (1.4%) | - | 1 (1.4%) |
| Biliary Tract (n=1) | - | - | 1 (1.4%) |
| Leukemia (n=1) | 1 (1.4%) | - | - |
| Liposarcoma (n=1) | - | - | 1 (1.4%) |
| **Total** | **14/17 (82.4%) primary cancer types** | **9/17 (52.9%) primary cancer types** | **11/17 (64.7%) primary cancer types** |
| Cancer stage | | | |
| Advanced/metastatic (n=50) | 24 (34.3%) | 17 (24.3%) | 14 (20.0%) |
| Non-metastatic (n=4) | 3 (4.3%) | 1 (1.4%) | 1 (1.4%) |
| Both metastatic/non-metastatic (n=7) | 4 (5.7%) | 2 (2.9%) | 2 (2.9%) |
| Relapsed/refractory (n=5) | 5 (7.1%) | - | - |
| Not reported (n=4) | 2 (2.9%) | 1 (1.4%) | 2 (2.9%) |
| **Total** | **38 (54.3%)** | **21 (30.0%)** | **19 (27.1%)** |
| *Note: some trials reported on more than one type of broad TTD definition, hence the total number of reports exceeds the number of reviewed trials.* | | | |

| Table A.6. Time to deterioration (TTD) definitions identified in the review | | | |
| --- | --- | --- | --- |
| **Broad TTD definition** | **Deterioration event definition** | **Number of endpoints (% of 849)** | **Number of trials (% of 70 trials)** |
| **TTCD** | **Confirmed** | **194 (22.9%)** | **21 (30.0%)** |
|  | Confirmed without death as an event | 77 (9.1%) | 14 (20.0%) |
|  | Confirmed with death as an event | 117 (13.8%) | 8 (11.4%) |
|  | Confirmed with PD as an event | 26 (3.1%) | 2 (2.9%) |
| **TUDD** | **Definitive** | **200 (23.6%)** | **19 (27.1%)** |
|  | Definitive without death as an event | 105 (12.4%) | 12 (17.1%) |
|  | Definitive with death as an event | 95 (11.2%) | 7 (10.0%) |
|  | Definitive with PD as an event | 46 (5.4%) | 7 (10.0%) |
| **TTFD** | **First** | **422 (49.7%)** | **36 (51.4%)** |
|  | First without death as an event | 350 (41.2%) | 30 (42.9%) |
|  | First with death as an event | 72 (8.5%) | 8 (11.4%) |
|  | First with death as an event using BPS | 24 (2.8%) | 1 (1.4%) |
|  | First with PD as an event | 15 (1.8%) | 2 (2.9%) |
|  | **First (implied)^1^** | **9 (1.1%)** | **2 (2.9%)** |
|  | First without death as an event | 9 (1.1%) | 2 (2.9%) |
|  | First with death as an event | 0 (0%) | 0 (0%) |
|  | First with death as an event using BPS | 0 (0%) | 0 (0%) |
|  | First with PD as an event | 0 (0%) | 0 (0%) |
| **Overall with death as event** | | **308 (36.3%)** | **21 (30.0%)** |
| **Overall with PD as event** | | **87 (10.2%)** | **11 (15.7%)** |
| **Overall with death and PD as an event** | | **50 (5.9%)** | **4 (5.7%)** |
| *All event definition used baseline as reference score unless otherwise specified. BPS; Best previous score (used as reference score). PD; Disease Progression*  *^1^ Definition not explicit in the paper* | | | |


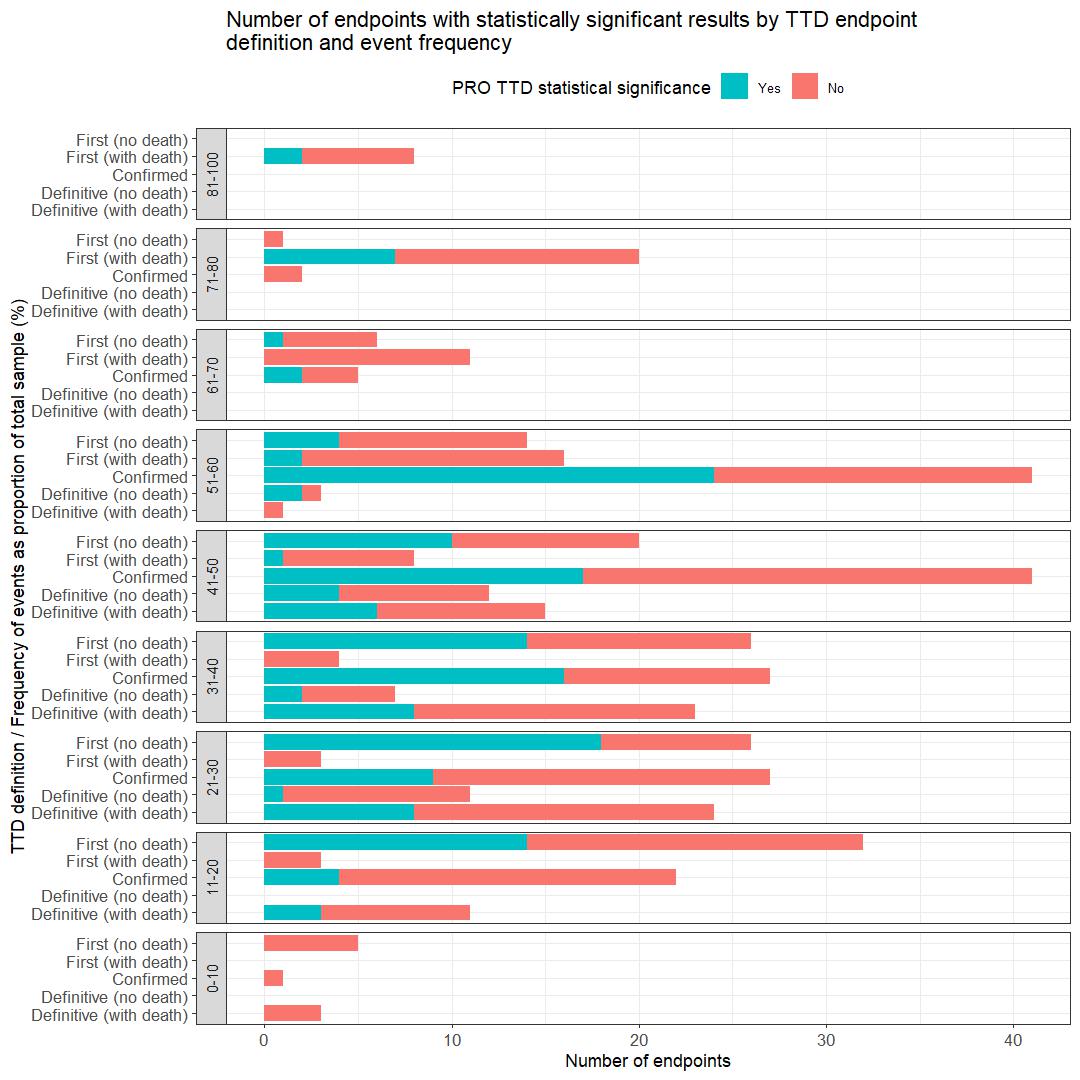


**Figure A.1. Proportion of Patients with Events by TTD definition and statistical significance**


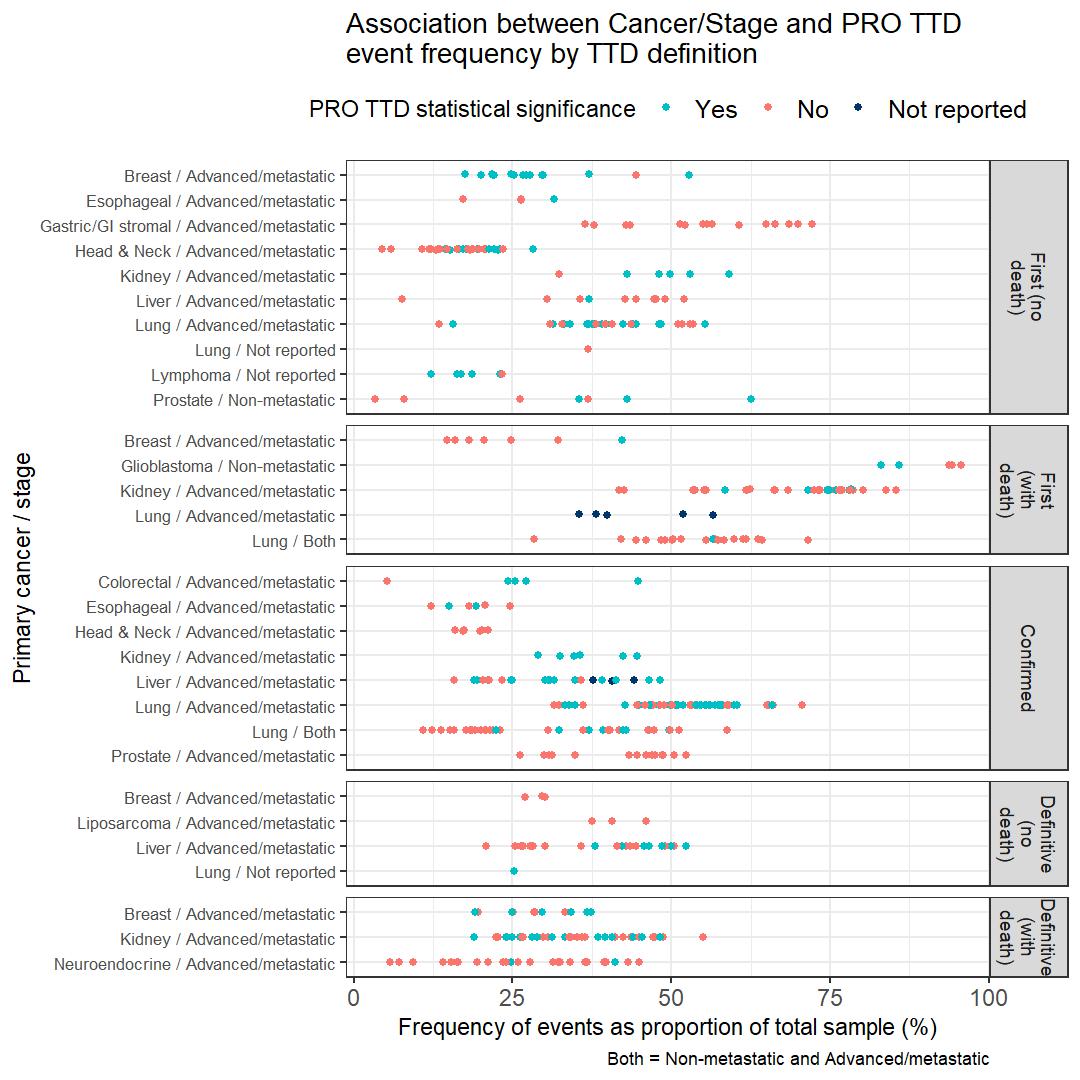


**Figure A.2. Proportion of Patients with Events by Cancer and TTD definition (TTD Statistical Significance in colour)**
